# A Unified Framework for Complex Networks with Degree Trichotomy Based on Markov Chains

**DOI:** 10.1038/s41598-017-03613-z

**Published:** 2017-06-16

**Authors:** David Shui Wing Hui, Yi-Chao Chen, Gong Zhang, Weijie Wu, Guanrong Chen, John C. S. Lui, Yingtao Li

**Affiliations:** 1 Huawei Technologies Co. Ltd., Hong Kong, China; 2 City University of Hong Kong, Hong Kong, China; 3 The Chinese University of Hong Kong, Hong Kong, China; 4 Huawei Technologies Co. Ltd., Shenzhen, China

**Keywords:** Information theory and computation, Complex networks

## Abstract

This paper establishes a Markov chain model as a unified framework for describing the evolution processes in complex networks. The unique feature of the proposed model is its capability in addressing the formation mechanism that can reflect the “trichotomy” observed in degree distributions, based on which closed-form solutions can be derived. Important special cases of the proposed unified framework are those classical models, including Poisson, Exponential, Power-law distributed networks. Both simulation and experimental results demonstrate a good match of the proposed model with real datasets, showing its superiority over the classical models. Implications of the model to various applications including citation analysis, online social networks, and vehicular networks design, are also discussed in the paper.

## Introduction

Many complex network models^1, 2^ have been proposed to provide an essential macroscopic understanding of various complex real-world networks^3–6^. One important feature is the node-degree distribution^4, 5, 7^, which belongs to two major classes: one is Poisson or exponential distribution (mainly for homogeneous networks, with rapidly decaying tails in the degree distributions); the other is power-law distribution (mainly for heterogeneous networks, well known for their scale-free properties, with long tails in the degree distributions). Existing models typically account for Poisson^8^ or exponential distribution^9, 10^ by the random attachment mechanism^11^ in network formation process, while power-law distribution comes from the preferential attachment mechanism^11–13^. Although both mechanisms are essential and to a certain extent, can capture many real-world phenomena, typically each of them works only within a particular range of the broad degree-distribution spectrum. In this work, we analyze a number of real datasets are analyzed, all exhibiting a “trichotomy” phenomenon — a power-law distribution in the middle range of the distribution with exponential-alike distributions in both the head and the tail regions. Thus, the two typical distributions, though fundamental, are not sufficient to individually provide an accurate fit to the entire range of degree distributions.

From a theoretical point of view, the study of accurate degree distribution generation mechanisms, including the trichotomy phenomenon^1, 14^, are recognized as an important and developing subject in complex networks study, as presented in some recent research monographs^1, 2^. Driven by application needs, recent network generation mechanisms include (1) a similarity attachment mechanism^15^ proposed to capture large-scale evolution of technological, social and metabolic networks, (2) exponential network generation mechanism proposed for (smart) power grids^10^, and (3) network cosmology approach to model causality for more realistic citation networks^16^. In particular, the idea of network organization is recently introduced to explain why some network (degree) distribution is closer to power law^17^, while some other networks exhibit a clear “trichotomy” distribution as presented in datasets of important real-world networks^14^, specifically the higher structural level of preferential attachment implies the less “trichotomy” the distribution will be. Besides giving better physical interpretation, new generation mechanisms also lead to better models for statistic estimation and testing^18^. Recent examples include estimation of degree distribution under fitness model^19^, and under exponential random graph model^20–22^ respectively, and estimation on other metrics, such as link prediction^23^. It also influences the recent development of other theoretical aspects such as controllability of complex networks^24^. It was found that the number of driver nodes to control a network is determined mainly by the degree distribution. Recent works^25, 26^ claim that node dynamics are also part of the determination, which is also strongly related to network formation. More related works have been reviewed in a recent overview article^27^. However, there is no unified generation method that can accurately capture all observed degree distributions, making it difficult to accurately estimate graph parameters or to precisely control complex networks.

From an application point of view, degree trichotomy, characterizing degree distribution properties into three regions, is a common way of analyzing different parts of citation distribution in both classical literature^28^ and recent literature^17, 29^. Despite its long history, a systematic and quantitative choice of regime parameters is still missing. Furthermore, many phenomena discussed in a complex network independently, could be unified in a degree trichotomy framework – small degrees (intial phase), medium degrees (middle phase), and large degrees (mature phase). For example, in the citation network, the first citation distribution and the issue of uncitedness^30^ of a paper could be viewed as a study on its initial phase. The cumulative advantage property (also called the preferential attachment effect)^31, 32^ is related to its middle phase. The aging property of a paper^16, 32–35^ could be related to its mature phase. Thus, a unified framework, or better off, a physically interpretable network generation mechanism, on citation network, unifying the above citation practice phenomena could be an important complement to recent alternative citation pattern generation models^36, 37^. Beyond citation analysis, an accurate characterization of degree distribution could also be of interest to recent developing networks such as online social networks^38, 39^ and Internet of Things (IoT)^40^. Specifically, in the network analysis, they utilize various degree distribution-dependent graph metrics, such as various centrality measures, clustering coefficients, and network diameters, for community detection and information disssemination speed estimation. In this paper, one will see that the proposed Markov Chain (MC) model can provide a quantifiable “degree trichotomy” and can serve as a better model on the aforementioned applications compared to existing models.

Besides having a major impact on the degree distribution modeling and consequently on the important measures of complex networks, “degree trichotomy” could be regarded as an universal property driven by some underlying physical mechanism. In a recent literature^17^, scale-free property of complex network is explained as a universal property, and driven by preferential attachment mechanism in the level of communities, i.e., a new node is preferentially attached to a finer grained organization structure (according to its size) instead of simply an arbitrary node or a link. This is called structural preferential attachment^17^. In this paper, we propose another attachment scheme that can produce trichotomy as a universal property of the network, with corresponding physical interpretations. In particular, the quantified “degree trichotomy” under our model provides an important criterion for determining the phase-transition points of complex networks, and this can be meaningful in particular applications (e.g., refining h-index in a citation network). Regarding the wide observations of many datasets exhibiting trichotomy in degree distributions^7, 17, 41, 42^, some models choose to focus on the power-law middle phase neglecting the other phases, while some models attempt to address the problem from its special facets. Those special facets are usually scenario-specific and a technical comparison of the proposed framework with those existing models are presented in Sec. S1 in the Supplementary Information (SI). The major issue of those existing attempts is still the lack of a general forming mechanism that can lead to a closed-form degree distribution exhibiting all three-phases with clear physical meanings. Such absence hinders further analysis on important measures of such networks. Furthermore, there were also two undiscussed practical issues in existing network generation models – initial settings and burst occurrence in node dynamics. Though neglected in most literature, these issues naturally arise in practical applications, and we will discuss them in the model description subsection.

In this paper, we propose a new framework based on Markov chains, to capture network formation processes, derive closed-form degree distributions and design a corresponding statistical test with estimated parameters having clear physical meanings. As examples, it will examine several respective complex networks: a classical example – citation networks, and two recent examples – online social networks and vehicular networks, to illustrate the applications of the proposed framework. In the SI, it will provide expressions of degree distribution (in both probability density form (in Sec. S3.6) and cumulative distribution form (in Sec. S6)), network diameter (in Sec. S7), and z-transform of degree distribution (in Sec. S5) (useful for deriving various graph metrics^43^) under the proposed network generation mechanism, which could be useful for subsequent network analysis. The proposed new network formation mechanism could also have potential applications in serving for more realistic models in new-born areas like econophysics and sociophysics, where the research is mainly based on scale-free network models.

## Results

In this section, a unified model is proposed based on Markov chains, referred to as the *MC model* hereafter, for representing the evolution process in a complex network.

### The proposed model – the MC model

#### Model description

The complex network is modeled as an evolving network, with nodes as its basic constituting component, and their degrees as the major characteristic of the network. The network starts with a few (connected) nodes and then evolves as follows.

New node comes to the network randomly at a certain rate; upon arrival, it creates zero, one or several connection(s) to existing nodes, and assume its starting degree (s. d.) *i* has probability mass *p*_*i*_(0).

Existing nodes have different degrees. As the network evolves, the degrees of existing nodes can change. The change of degree of a particular existing node is divided into three phases — initializing, fast-evolving, and maturing phases.

**Phase 1** – **Initializing:** All nodes with a degree less than or equal to parameter ${\mathcal L}$ are called in the initialization phase. Such nodes have a uniform, low attractiveness, *i*.*e*., their evolution is independent of the structure of the current network.

**Phase 2** – **Fast-evolving:** All nodes with a degree larger than parameter ${\mathcal L}$ but less than or equal to parameter ${\mathcal{U}}$ are called in the fast-evolving phase. Their attractiveness is proportional to their degree, *i*.*e*., when a new node comes, it prefers connecting to an existing node of a higher degree. In this phase, their evolution depends on the existing network structure. This preferential attachment mechanism enables some nodes to become more and more popular as the network evolves.

**Phase 3** – **Maturing** (**Saturating**)**:** All nodes with a degree larger than parameter ${\mathcal{U}}$ are called in the maturing phase. Their attractiveness begins to saturate due to physical, economical or technological constraints. These nodes with degrees exceeding a certain threshold are called super nodes. Although new nodes still preferentially join the super nodes, they join these super nodes with the same probability. There are two reasons. One is that existing super nodes could start to refuse new connections due to physical or economical constraints. The other is that although super nodes differ in their degree, new nodes joining the network will not care or cannot distinguish the exact degrees of those super nodes. In both cases, a new node will attach to the super nodes with (approximately) the same probability. Thus in the maturing phase, the evolution of the information consumption pattern is still dependent on the existing network structure, but the dependence becomes weaker as the network becomes larger.

Occasionally, instead of independent increment, several nodes simultaneously come to connect to an existing node in the network due to certain special characteristic (degree) of the node is attached.

**Burst-attractive:** A node with a degree equal to *k* ^*b*^ is called a burst-attractive node. When a node reaches this characteristic degree *k* ^*b*^ of the network, then it can attract multiple new nodes simultaneously.

**Physical meanings of parameters:** Other than the meaning of *k* ^*b*^ mentioned above, the general meanings of other parameters introduced and some extra parameters are also included as follows:

The parameters ${\mathcal L}$ and ${\mathcal{U}}$ represents the thresholds on the strengths (in terms of degree) to trigger the start and the end of fast-growing phase. In the mathematical model described later, we also introduce extra parameters *λ*, *L* and *U*, and *γ*, which serves as the parameters related to the mean arrival rate of the node, and the strengths (in terms of degree) of the attachment before the start and the end of fast-growing phase, and also the speed of fast-growing phase respectively. All these parameters could be viewed as characteristics of the structure of the internal organization of the network^17^.

Two examples are given as follows to illustrate the application of the proposed model.

**Example 1: Citation Networks**. In citation networks, *k* ^*b*^ is the number of citations for a paper to be first considered as important. This number could depend on the convention of the research field, or common authors and journals research advertisement practice. One may view *k* ^*b*^ citations as the time a paper is first recognized as “sleeping beaulties” in citation analysis jargon^32^. *p*_*i*_(0) is the probability that the number of citations is *i* when a paper is being published (i.e., time 0). So most of the probability mass is at *p*_0_(0), but some probability could be at *p*_*i*_(0) for small *i*, due to some related works at similar time but published earlier, and cited this paper before it is published. ${\mathcal L}$ and ${\mathcal{U}}$ could serves as the two separating boundaries of the “degree trichotomy” in citation distribution mentioned in the introduction. Besides that the model parameters *γ*, *L*, ${\mathcal L}$, ${\mathcal{U}}$ are designed to capture several important phenomenon discussed in citation analysis literature. Details are presented in Methodology section.

**Example 2: Online Social Networks**. In online social networks, *k* ^*b*^ is the number of friends such that a person is first considered as celebrity causing sudden burst of attraction, or friend recommendation engine starts to have enough data to attract a burst of new comers to add this person as friend simultaneously. *p*_*i*_(0) is the probability that the number of initial friends is *i* when a person just join the online social network, the probability mass could also spread over for several *i*.

It is noted that both examples can be regarded as information consumption processes. For example, in citation networks, if paper A is related to a newly written paper B, then the author(s) of paper B has some probability to get to know paper A and thus cite it. We call this incident as an information consumption attempt. Once paper B cites paper A (or the attempt is successful), then the author(s) of another paper C may note paper B and cite it. This shows how this sequence of attempts can connect to each other, which is viewed as the connection of citation networks. Similar explanations can be formulated for social networks.

#### Mathematical formulation (The Markov Chain (MC) Model)

In this section, a network model is formulated mathematically. Consider a connected network is initialized with certain number of initial nodes with arbitrary initial connections between them (e.g., the simplest possible initial network is a chain with only two nodes connected by one edge). As time evolves, new nodes arrive, and each of them would connect to an existing node with certain probability, which depends the on the phase the existing node is in, described as follows.

When the network size (the number of nodes) is *n* − 1, and a new node arrives, it will connect to the *i*-th existing node, *i* ∈ {1, … n − 1}, with a probability proportional to a constant ${\hat{k}}_{i}^{(n-\mathrm{1)}}$, which we call the modified degree. Denote ${{\hat{k}}_{i}}^{(n)}$ by $\widehat{{k}_{i}}$ and ${k}_{i}^{(n)}$ by *k*_*i*_, whenever the corresponding network size *n* is specified. According to the three phases we defined before, the modified degree can be expressed as (with $\widehat{-1}\triangleq 0$):

1 $${\hat{k}}_{i}=\{\begin{array}{cc}L, & \text{if}\,0\le {k}_{i}\le {\mathcal{L}}\\ {k}_{i}, & \text{if}\,{\mathcal{L}} < {k}_{i}\le {\mathcal{U}}\\ U, & \text{if}\,{\mathcal{U}} < {k}_{i}\le {N}_{{\mathcal{T}}}\end{array}.$$

where ${N}_{{\mathcal{T}}}$ is the network size at current observation time ${\mathcal{T}}$, and typically *L* and *U* are restricted to $L\le  {\mathcal L}$ and ${\mathcal{U}}\le U$.

To enable subsequent mathematical analysis, we consider a specific stochastic model as follows. When the network size is *n* − 1 at time *t*, in a small time interval (*t*, *t* + Δ*t*), there is a probability $\lambda {\rm{\Delta }}t{\hat{k}}_{i}$ that some new node(s) arrive to the network and connect to node *i*, and the remaining probability $(1-(\lambda {\rm{\Delta }}t\sum _{i=1}^{n-1}{\hat{k}}_{i}))$ would be no node arrival. In most cases, when there are new node(s) to be attached to node *i*, it would be the case with only one single node arrival, but when node *i* reach certain degree *k* ^*b*^, bursty arrivals to it could happen, i.e., several new nodes can simultaneously connect to node *i* create a burst increase in its degree *k*_*i*_. Specifically the probability of increasing from degree *k* ^*b*^ to degree *k* within this small time interval Δ*t* would be $\lambda {\rm{\Delta }}t{\hat{k}}_{i}{p}_{{k}^{b}\to k}$. Under this model, the network formation process is a Markov Chain with the state variable completely characterized by the network size *n* and the degree *k*_*i*_ of each node *i*, with the following state evolution equation.

Denote the probability mass function of the degree of any specified node * by ${p}_{k}(t)=Pr\{K(t)=k\}$. According to the derivation in the methodology section, its degree dynamics is simplified as:

2 $$\begin{array}{rcl}\frac{d}{dt}{p}_{k}(t) & = & \lambda (\widehat{k-1}){p}_{k-1}(t){\mathbb{1}}(k\ne \{\mathrm{0,}{k}^{b}+1\})\\  &  & +\lambda (\widehat{{k}^{b}}){p}_{{k}^{b}\to k}{p}_{{k}^{b}}(t){\mathbb{1}}(k\ge {k}^{b}+1)-\lambda (\hat{k}){p}_{k}(t),\end{array}$$

where ${\mathbb{1}}(\cdot )$ is the indicator function.

**Figure 1:**
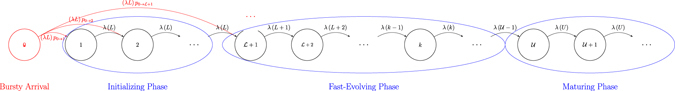
The state transition rate diagram of the MC for each particular arrived node (i.e. dynamic eq. (2) with trichotomy model eq. (1)), with its degree *k* as the state variable (with the burst occurred at *k* ^*b*^ = 0 as an illustration). The blue part represents the usual state transition (or developement) of the degree having trichotomy in its transition rate $\lambda \hat{k}$ according to its phase (wherein the transition is triggered by a single new node arrival). The red part represents the burst occurrence, i.e., some new node(s) coming at a rate of *λL* simultaneously, with the rate of making *i* connections simultaneously with this existing node being *λLP*_0 →*i*_. Noted that the initial distribution of the degree of this arrived node is denoted as *p*_*i*_(0) for all degree *i*, is not shown in the figure.

The state transition rate diagram of the degree of the specified node is shown in Fig. 1 (with *k* ^*b*^ = 0 as an example).

Now, one is ready to analyze the node degree distribution *p*_*k*_. Beside deriving *p*_*k*_(*t*) from eq. (2), one also needs to know how long (i.e., the residential-time, *T*) the specified node * has been staying in the network. Suppose that the network starts at time 0, and node * arrives at time t_*_. Then, at an observation time ${\mathcal{T}}$, the residential-time *T* of node * is ${T}_{\ast }={\mathcal{T}}-{t}_{\ast }$. Denote the corresponding distribution as *f*_*T*_(*t*). This distribution depends on the differential-difference equation for the marginal distribution of *N*(*t*), obtained by summing eq. (4) over *k*. Then ${p}_{k}={E}_{T}[{p}_{k}(t)]$. Detailed calculations are presented in the Sec. S4 of the SI.

### Theoretical Results

Mathematically, we prove that the proposed MC model is a generalization of several existing models such as the Poisson network model, exponential network model, and BA model, by appropriately setting the physical constraint-related parameters: lower bound *L*, lower threshold ${\mathcal L}$, upper bound *U*, and upper threshold ${\mathcal{U}}$; and generally, the MC model can deduce the trichotomy in degree distribution. The results are stated as several theorems as follows with a sketch of proof of the general case in appendix. Details of all proofs are also provided in Sections S3 and S4 of SI.

**Remark 1** The model can also be extended to directed/undirected cyclic/acyclic networks. When a node joins the network, it can be a bursty arrival and associate its multiple edges with existing nodes, Each edge could be directed or undirected. With multiple (un)directional edges added, the resultant network could be directed/undirected cyclic/acyclic. By keeping track of the state variables in in- or out-degrees, we can perform similar analysis as in the case of degrees for undirected graphs.

In the following, we denote the MC model with no burst structure (i.e., ${p}_{{k}^{b}\to k}=\mathrm{0,}\forall k\ge 1$ except ${p}_{{k}^{b}\to {k}^{b}+1}=1$) and the same starting degree *k* ^*s*^ for nodes (i.e., ${p}_{k}(0)=\mathrm{0,}\forall k\ge 0$ except ${p}_{{k}^{s}}(0)=1$) as “the default MC model”.

#### MC generalizes the Poisson network model and exponential network model

**Theorem 1** *When* $L= {\mathcal L} ={\mathcal{U}}=U$ *in the default MC model*, *as* ${\mathcal{T}}\to \infty$, it reduces to one of the two classical models respectively, for

- Case 1 — accounting a fixed set of nodes (starting at the same time and degree): it reduces to the Poisson network model;
- Case 2 — accounting all nodes: it reduces to the exponential network model.

**Proof** Proof of theorem 1 is given in Sec. S3.4 of the SI.□

#### MC generalizes the power–law model

**Theorem 2** *When* $L= {\mathcal L} =1$, $U={\mathcal{U}}=\infty$ in the default MC model, as ${\mathcal{T}}\to \infty$, it reduces to the power-law model.

**Proof** Proof of theorem 2 is given in Sec. S3.5 of the SI.□

**Remark 2** When the MC model is equipped with non-singleton starting degree or with burst structure, theorem 1 and theorem 2 can be generalized to give mixture of those classical distribution, such as mixture of exponential or power–law (giving power–law with exponent slightly greater than −3). Specifically, with non-singleton starting probability, ${p}_{k}={\sum }_{i=0}^{k}{q}_{i}{p}_{ki}{d}_{ki}$, where *q*_*i*_ = *p*_*i*_(0) is the mixing probability, *p*_*ki*_ is the probability mass of classical law with starting degree *i*, and *d*_*ki*_ = 1. More generally, with the burst structure, *d*_*ki*_ is a discounting factor (<1), and *q*_*i*_ is a more general mixing probability.

#### MC explains observed trichotomy in General Case

In Experimental Results section below and Sec. S8 of SI, experimental results in different network datasets are presented. Such examples exhibit three phases in their degree distributions, and we conjecture this phenomenon could be general in nature. The proposed MC model offers an analytical closed-form expression of the degree distribution and can explain the observed three phases in empirical degree distributions in real networks. The results are summarized as follows:

**Theorem 3** The degree distribution of the default MC model (with starting degree *i*) in general parameter settings is given by

3 $${p}_{ki}\sim \{\begin{array}{ccc}c\cdot {\mathsf{g}}{\mathsf{e}}{\mathsf{o}}{\mathsf{m}}(\frac{\gamma }{\gamma +L}) & (\text{with s.d.}=i), & \text{if}\,0\le k\le {\mathcal{L}}\\ {c}_{2i}\cdot {\mathsf{p}}{\mathsf{o}}{\mathsf{w}}{\mathsf{e}}{\mathsf{r}}\mbox{-}{\mathsf{l}}{\mathsf{a}}{\mathsf{w}}\,\text{with exponent}-(\gamma +1) & (\text{with s.d.}={k}_{2i}^{s}), & \text{if}\,{\mathcal{L}} < k\le {\mathcal{U}}\\ {c}_{3i}\cdot {\mathsf{g}}{\mathsf{e}}{\mathsf{o}}{\mathsf{m}}(\frac{\gamma }{\gamma +U}) & (\text{with s.d.}={k}_{3i}^{s}), & \text{if}\,{\mathcal{U}} < k\le {N}_{{\mathcal{T}}}\end{array}$$

and the degree distribution of the general MC model is a mixture of the above distributions over various *i* with mixing probability *q*_*i*_ and burst related discount factor *d*_*ki*_ as ${p}_{k}=\sum _{i=0}^{k}{q}_{i}{p}_{ki}{d}_{ki}$, where *L* ≤ *γ* ≤ *L* + 1. For $U\ll N$, one has *γ* ≈ *L*, while for *U*~*N*, one has *γ* ≈ *L* + 1;

*c*, *c*_2*i*_, *c*_3*i*_ are normalization constant making the total probability to be 1, i.e., ${\sum }_{k}{p}_{k}=1$;

$$({c}_{2i},{k}_{2i}^{s})=\{\begin{array}{cc}(p{\mathcal{L}}i\cdot \frac{L}{\gamma },{\mathcal{L}}+1) & \text{if}\,i\le {\mathcal{L}}\\ (c,i) & \text{if}\,i > {\mathcal{L}}\end{array}$$ and $$({c}_{3i},{k}_{3i}^{s})=\{\begin{array}{cc}(p{\mathcal{U}}i\cdot \frac{{\mathcal{U}}}{\gamma },{\mathcal{U}}+1) & \text{if}\,i\le {\mathcal{U}}\\ (c,i) & \text{if}\,i > {\mathcal{U}}\end{array}$$ are the (normalization constant, starting degree) pairs for degrees at phase 2 and phase 3 (It is noted that when s.d. is outside the boundary of the phase, the probability mass function is defined as 0 at that phase.); ${N}_{{\mathcal{T}}}$ is the network size at current time ${\mathcal{T}}$.

With no burst structure, *q*_*i*_ = *p*_*i*_(0), i.e. the starting degree distribution, and *d*_*ki*_ = 1.

With burst structure, *q*_*i*_ is greater than *p*_*i*_(0) by a factor proportional to the probability of transfer from *k* ^*b*^ to *i*, i.e., ${d}_{ki}={p}_{{k}^{b}\to k}$ when *k* ^*b*^ is in the interval between *i* and *k* − 1; and *d*_*ki*_ = 1 otherwise.

**Proof** Proof of theorem 3 are sketched in the Appendix and are given in details in Sec. S3.6 of the SI.

**Remark 3** The general solution of the degree distribution under the MC model without the trichotomy structure (eq. (1)) could also be found as given in Sec. S3.3 of SI. Further analysis, e.g., Z-Transform (in Sec. S5) and the cumulative distribution function (cdf) (in Sec. S6) of the network degree distribution, and network diameter (in Sec. S7), under the MC model are also derived respectively in the sections S5, S6, and S7 of SI. These results not only can show the impact of new system parameters, they can also have profound impact in deriving other graph metric^43^, other fitting method based on cdf, and analytic tractable general diameter formula in contrast to simulation-based power-law counterpart result^7^.

### Simulation Results

The node-degree distribution of the MC model has been simulated, for a network of *N* = 100,000 nodes. The simulation is set up according to the model as follows: there is a certain initial set of nodes of degree 0 at the beginning of each time. When a new node comes, it will connect to an existing node according to our MC model. When there are 100,000 nodes, the empirical node-degree distribution of the focused group of nodes is reported. For generating the Poisson model under the MC model, the focused group is the initial set of nodes. In that simulation, we set 50,000 initial nodes which is enough for demonstrating the statistics. For other networks, the focused group is all the nodes in the networks. In that simulation, the initial size is 1 (just for the sake of matching the literature convention in generating those networks). It is remarked that this setting does not lose any generality, because according to our theorem of our proposed framework, same statistics properties could be also be generated for different initial sizes. Every simulation is repeated for 100 times, and the average result is reported.

**Figure 2:**
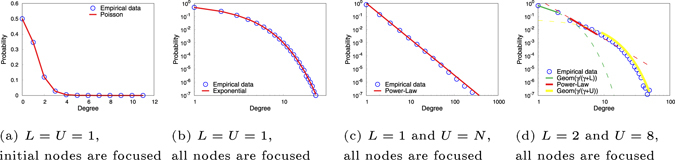
Simulation results of node-degree distribution on the MC model with varying *L* and *U*. The blue circle points represent the empirical probability density function (PDF). The red solid line represents the theoretical prediction of the PDF. In (**c**,**d**), the empirical PDF was plotted using logarithmic binning to avoid over-fitting due to large variations in the tails.

First, by setting $L= {\mathcal L} =U={\mathcal{U}}=1$ in the MC model, when the focused group is the initial set of nodes, it reduces to the Poisson model. The results are shown in Fig. 2(a); when the focused group is the all the nodes, it reduces to the exponential network model, with results shown in Fig. 2(b). Second, by setting $L= {\mathcal L} =1$ and $U={\mathcal{U}}=N$, the MC model reduces to the BA model. The results are shown in Fig. 2(c).

Finally, to show a general scenario, by setting $L= {\mathcal L} =2$ and $U={\mathcal{U}}=8$, the MC model generates the trichotomy distribution shown in Fig. 2(d), i.e., a power-law distribution with exponential head and tail. Several characteristics of the node-degree distribution plot matches with those predicted by theory. The exponent of the power-law region, −(*γ* + 1), depends on *L*: specifically, $L\le \gamma \le L+1$, particularly for small *U* as in this case, *γ* ≈ *L*. In Fig. 2(d), *L* = 2, it matches with the observed exponent *γ* + 1 = 3. It is also observed that the head part is geometrically distributed with parameter 0.6 and the tail part is geometrically distributed with parameter 0.27. Several other simulation results under different system parameters setting on *L* and *U* are presented in the Sec. S8.1 of SI and the relationship between the observed variation of the node-degree empirical distribution of the simulated data and these system parameters are also further discussed in the Sec. S8.2 of SI.

### Experimental Results

The trichotomy behaviour from real-world networks, including citation network, social networks and vehicular networks, are studied in this article, via nine real-world datasets. Citation network is the major example of applications of the MC model in the main document, for other networks, the experimental results are presented in Sec. S10 of SI.

In this section, three datasets from scientific publication citations and patent citations^7, 41, 42^ are studied, as shown the following Table 1. They are used to demonstrate a trichotomy behaviour in the node-degree distribution of citation networks, where node-degree in this case corresponds to the number of citations for each dataset. Those results are given in the Sec. S10 of SI. In each type of real-world network of this section and section S9 of SI, the corresponding physical meaning of the Information Consumption Model (ICM) of that network, the parameters estimation (i.e., the fitting) of the proposed model for that network from the real-world datasets according to our proposed procedure (described in Sec. S9 in SI), and the utility of the ICM of that network are provided in the corresponding section.

**Table 1:** Citation Datasets.

Dataset	Date	#Nodes
DBLP Citation^41^	1995–2014	2,146,341 papers
APS Citation^42^	1893–2013	531,478 papers
US Patent Citation^7^	1975–1999	3,774,768 patents

#### Empirical Data and Fitting Results

**Figure 3:**
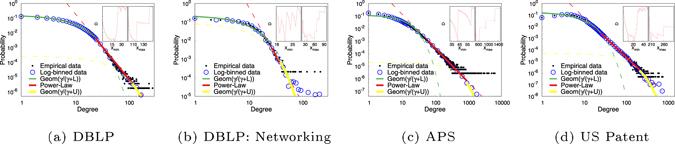
Citation probability versus the number of citations on a double logarithmic scale using partial logarithmic binning^57^. The inner figures show the values of Kormogorov-Smirnov test vs. *X*_*min*_ and *X*_*max*_. ${\mathcal L}$ and ${\mathcal{U}}$ are selected from *X*_*min*_ and *X*_*max*_ to minimize the KS-test distance value. The detail of the fitting method is presented in the Sec. S9 of SI.

Figure 3 shows the distributions of citations for DBLP Computer Science publications^41^, American Physical Society (APS) publications^42^ (also studied previously^44^ with older dataset), and US patents datasets^7^. Since some recent researches^45^ show there are significant difference in citations impacts for the case within on field of research and the case for interdisciplinary research (IDR), we conduct our analysis from degree distribution viewpoint (which is different from the literature^45^) in a subset of papers on computer networks in DBLP datasets as shown in Fig. 3(b), in comparison with the full DBLP database that contain some interdisciplinary research. We observe similar “trichotomy” behavior in terms of degree distribution, yet there are also some characteristic differences in terms of the estimated parameter as shown in Table 2, leading to a supportive result to the literature^45^, i.e., IDR leads to higher citation impact as discussed below.

**Table 2:** Fitting parameters and errors in citation datasets.

Dataset	${\mathcal L}$	${\mathcal{U}}$	exponent	*MC*	*PL*	*PL* − *C*	*SPL* − *C*
DBLP	20	118	−4.93	0.02	0.81	0.51	0.18
Networking	21	39	−4.05	0.06	0.83	0.59	0.30
APS	43	652	−2.69	0.02	0.62	0.50	0.02
US Patent	22	213	−3.35	0.13	0.82	0.70	0.20

*MC* is the fitting error of our model, *PL* is the fitting error of using power-law, *PL* − *C* is the fitting error of using power-law with expoenetial cutoff, and *SPL* − *C* is the error of shifted power-law with geometric cutoff.

We compare our MC model with three other models, power-law $({p}_{x}={x}^{-\alpha })$, power-law with exponential cutoff $({p}_{x}={x}^{-\alpha }{e}^{\beta x})$, and shifted power-law with geometric cutoff model^46–48^ $({p}_{x}={(x+{x}_{0})}^{-\alpha }{{\rm{\Omega }}}^{x})$. We use Kolmogorov-Smirnov statistic to quantify the error between the fitting curve and the empirical data. The fitting parameters and errors are shown in Table 2. Power-law has one estimated parameter in the model, power-law with exponential cutoff model has two, while MC model and shifted power-law with geometric cutoff model both have three parameters. One can see that our MC model out-performs these models by 34–97% in DBLP, DBLP-Networking, and US Patent datasets. In APS dataset, MC model performs similarly to the shifted power-law with geometric cutoff.

With the small fitting error, according to the discussion section, one may try to use the parameters ${\mathcal L}$, ${\mathcal{U}}$ and *γ* to draw some results on comparison citation practice in different areas on research. One can see that there is a larger ${\mathcal L}$ and ${\mathcal{U}}$ in APS than DBLP (a citation database for Computer Science (CS)) and US patent. It means a paper published in physics (in APS) has a longer time in “Sleeping Beaulties”, as well as more sensitive to high-cite article, compared to CS (which is also the case when compared the general CS with a restricted area, as one could also see a larger ${\mathcal{U}}$ in DBLP in general (with IDR) than the specific Networking area). A larger *γ* in DBLP than that in APS implies a faster increase in publications number, but slower increase in citation count. The smaller *γ* in APS could also be explained by burst structure (due to more notable awards in physics), causing simultaneous citation and leading faster accumulation according to the MC model. The larger *γ* and large ${\mathcal{U}}$, could mean CS (particularly a small field within CS, e.g., networking) is easier to get aware and more publications and is easier to get forgotten compared to physics. US patent has an effect in between CS and physics. It is remarked that in US patent, the fitting error is worst than other case. It is possibly due to more different starting degree and burst structure parameter could increase the fitting error. A possible cause is that there are more cross-discipline results in US patent, and hence more varieties in citation practice on starting degree, as observed in beginning few degrees in citation network distribution in Fig. 3(d), the default MC model which is used for result plotting of Table 2 is not yet enough to provide a perfect fit. Nevertheless, one could further improve the fitting error with the general MC model for introducing mixing probabilities from starting degrees support and burst structure.

## Discussions

Since the proposed MC model introduce many extra features compared to existing complex network models (elaborated in S1 of the SI), it could help us to infer many new characteristics of the real-world network where existing models failed. In this section, the citation network example is discussed to illustrate how the MC model can use experimental data in citation network to infer some new characteristics of the network for citation analysis usage. In the S9 of the SI, social network and vehicular network examples are illustrated. It is remarked that examples are mainly for illustration purpose, the MC model, as a unified framework of existing models, with new features, could also inspire explorations of new characteristics on other real-world networks, particularly those studied in the existing complex network literature^1–7^.

### Modeling the citation network using the MC model

In citation networks, publications (papers) are presented as nodes. When a new publication *p*1 cites another (existing) paper *p*2, a link is built between *p*1 and *p*2, and the node degree of *p*2 increases by one. In the MC model, the number of citations (i.e., links) that a new publication create is a random variable, specifically it creates *i* citation with probability *p*_*i*_(0). A special feature of the MC model compared to existing work, is that in the MC model, among those lowly cited paper (i.e., papers with citations $\le  {\mathcal L}$), a new paper is likely to cite any of them at random (with proportionality factor *L*). It models the effect that several new publications have equal chance of being sleeping beauties, instead of first mover advantage of “cumulative advantage”. When the citation count of a paper grows, citation once it reaches certain (degree) citation, *k* ^*b*^, researchers in that area will be aware of it (may be via journal website advertisement), once it is recognized as “Sleeping Beaulty” (i.e., its importance is recognized by fellow researchers), it will attract multiple citation simultaneously (noted that this burst arrival point *k* ^*b*^ could depend on research area citation practice). Other than *k* ^*b*^, as citation increases, there could be a common practice that when a paper get citation count $>  {\mathcal L}$ (i.e., the paper is already got certain level of citations), it will be viewed as part of the state-of-the-art results. New papers may choose to cite the paper with a higher probability when it has higher citations among those state-of-the-art papers. For those papers that are highly reputable (*i*.*e*., papers with citations >${\mathcal{U}}$), a new paper may cite any of them with equally high probability, which are all treated as “core documents”. A larger ${\mathcal L}$ in a research area means that a paper needs a larger number of citations before starting to draw significant attention, and a larger ${\mathcal{U}}$ means that the study in the area may have been very popular. The interval between ${\mathcal L}$ and ${\mathcal{U}}$ can represent the amount of work needed to be done to explore, verify, or extend a work so as to make it become one of the most reputed papers in its field. A larger *L* represents a larger initial attractiveness of a paper before becoming a state-of-the-art result of the field, and a larger *U* represents a larger saturated attractiveness when the paper became reputable.

There is a strong relationship between the parameters of the MC model and citation analysis in the existing literature. The MC model treats the citation count from 0 to ${\mathcal L}$ as the entry level or low cited regime (for modeling “uncitedness”). The regime of citation count from ${\mathcal L}$ to ${\mathcal{U}}$ is used to model the intermediate or cumulative advantage regime, and what beyond ${\mathcal{U}}$ is treated as the “highly cited”^29, 35^ or “citation classic”^49^ regime. Further corresponding meaning of the parameters in the MC model and their role as quantitative guidelines for subsequent citation analysis are given as follows.

### Illustration of physical meaning in citation networks

#### Meaning of *L*

The probability of uncitedness is shown to be related to the mean arrival rate of citations to a publication in existing citation models^50^. This feature is also captured in our model as both the probability of uncitedness (*p*_0_) and the mean arrival rate (*λL*) are related to parameter *L*.

#### Meaning of *γ*

In citation network statistics, the number of citations increases over year. In comparing the exponents of the power-law distributions, we could use the same interpretation provided by Redner^28^: a smaller power-law slope *γ* means that the database has higher citation count increment, or equivalently less publication number grow. In the MC model, *n* denotes the number of papers, and *λL* is the mean arrival rate of publication, with *L* is the normalized rate, so according to Redner^28^, *γ* is proportional to *L*, which is now theoretically established as a corollary of the trichotomy theorem in our MC model.

#### Meaning of ${\mathcal L}$

One can gain extra insight of the citation statistics based on our model parameters and network generation mechanism. The parameter ${\mathcal L}$ could be a simple yet effective new scientometric indicator, which helps one to judge whether a paper has already passed the most difficult time of attracting the first few citations. In the literature, there is an important concept called “sleeping beauties” (i.e., even an important paper takes time to outstand other papers)^31^, cumulative advantage (i.e., preferential attachment) mechanism has first mover advantage (i.e., favor those papers who publish earlier), so cannot generate “sleeping beauties” (which are important papers, but not necessarily published in an earlier time) reasonably. In contrast, our network generation mechanism provides fair chances of generating “sleeping beauties” (by using an equal rate, proportional to *L*, among those potential candidate nodes, i.e., those nodes before reaching degree ${\mathcal L}$). In other words, one does not need to look into the details of the degree distribution every time to make this judgment.

#### Meaning of *k*^*b*^

In the literature, once a “Sleeping beauty” is recognized, there will be a burst of citations associated to this “Sleeping beauty” simultaneously. In the MC model, this effect is modeled by each node reaching *k* ^*b*^ has a chance of being “Sleeping beauty”, and attract a burst of citations of different size (to reach citation count *k*) with different probability ${p}_{{k}^{b}\to k}$.

#### Meaning of ${\mathcal{U}}$

Similar to ${\mathcal L}$, the parameter ${\mathcal{U}}$ is a simple yet effective scientometric indicator to judge whether a paper has reached the aging stage, a well-recognized phenomenon in citation analysis literature.

With this improved citation network node attachment model, one could further combine it with the aforementioned recent modeling techniques (e.g. aging-related function and fitness function^32^) to capture the impact of a publication year, as well as “novelty” fitness^32^, to model the heterogeneity of being “beauties”, and then have a more accurate model of evolution of citation dynamics of a paper or a research topic.

#### Implications to citation analysis

The purpose of citation analysis is to understand citation behaviors, based on which researchers can design better scientometric (or bibliometric) indicators. These indicators may help in both managerial tasks (e.g., ranking scientists^51^, assessing journal importance^52^) and improving database consistency^53^) and communication tasks (e.g., researchers can easily obtain important/relevant information from others’ publications^54^). Studying the degree distribution is an important procedure of citation analysis, because it captures the important statistics of a research area in a particular database^55^. One typical example of the statistics is to design more reasonable scientometric indicators for ranking scientists. For example, a recent work^51^ proposea new index, called o-index, used to evaluate an individual researcher, which has several benefits over the traditionally known indices like h-index. However, we notice that the o-index only takes the highest paper citation count into consideration, but does not include the full observation of the degree distribution of a citation network. It may also suffer the same deficiency of non-robustness of h-index as pointed out in the literature^56^. The highest citation may be due to some random factors (e.g., the researcher co-authored a paper with a famous scientist, which leads to a single highly-cited paper). Merely relying on this count prohibits the o-index from a more accurate interpretation.

Using our model, one can utilize the distribution to construct a more reasonable index. One possibility is to replace the o-index (which is $\sqrt{mh}$, where *m* is the citation count of the researcher’s mostly cited paper, and *h* is his/her h-index, defined by first ordering his/her publications’ citation counts ${\{{c}_{r}\}}_{r=\mathrm{1,2,}\cdots }$ in descending order with *c*_*r*_ being the *r*-th one, and h-index is the maximum *r* with ${c}_{r}\ge r$) by $\sqrt{{n}_{{\mathcal{U}}}h}$, where ${n}_{{\mathcal{U}}}$ is the number of his/her papers in the regions whose citation counts are larger than a threshold (e.g., ${\mathcal{U}}$). Such a modification of o-index using our model parameter ${\mathcal{U}}$ inherits the benefit of o-index over h-index in ranking scientists in the citation network context (similar reason was explained^51^), while it is statistically more robust, as the effects of outliers and the highest citation count decrease.

## Methods

### Methods for Model formulation

**Figure 4:**
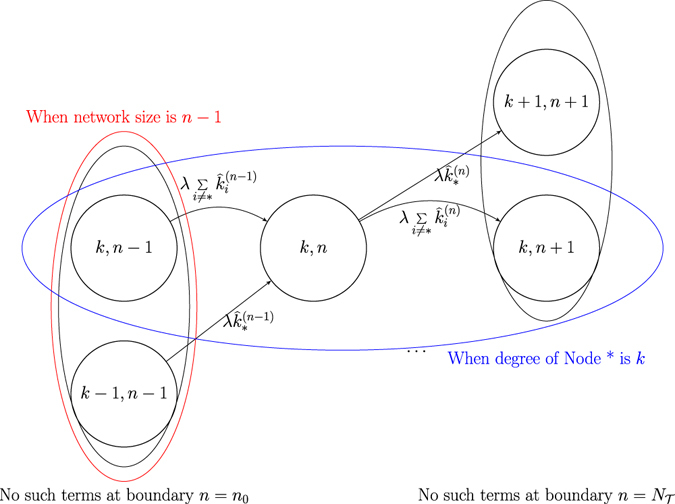
The state transition rate diagram of a two-dimensionalMarkov chain for a specific node *— where *k* denotes its degree and *n* denotes the network size that this node * is in, with values *n*_0_ and ${N}_{{\mathcal{T}}}$ respectively at its initial time and time ${\mathcal{T}}$. In the diagram, it is noted that ${\hat{k}}_{\ast }^{(n-1)}=\widehat{k-1}$ when ${k}_{\ast }^{(n-1)}=k-1$, and ${\hat{k}}_{\ast }^{(n)}=\hat{k}$ when ${\hat{k}}_{\ast }^{(n)}=k$.

In this section, the method to derive the essential individual degree dynamic eq. (2) is elaborated. From the MC model description, consider a specific node (denoted as node * hereafter), then its degree k_*_ and the corresponding network size *n* together would form a two-dimensional (continuous-time) Markov chain, with its state transition rate diagram depicted by Fig. 4 (for better illustration, the burst structure is not shown in this figure).

In Fig. 4, a circle represents a state of the state variable. Suppose that node * in consideration has degree *k* − 1 and the current network size is *n* − 1. Then, according to the modified preferential attachment mechanism, a newly arrived node connects to node * at rate $\lambda (\widehat{k-1})$. But, it is also possible for this new node to connect to other nodes. Since the new node is likewise preferentially attached to other nodes in proportion to their modified degrees, the total state transition rate of connecting to other nodes is given by $\lambda {\sum }_{i\ne \ast }{\hat{k}}_{i}$. In the following, consider that it connects to node *i*′.

Denote the number of nodes in the network at time *t* by *N*(*t*) and denote the degree of the specified node * at time *t* by *K*(*t*) as two random processes. Then, the joint probability mass (density) function of this node degree and the network size at any particular time *t* is ${p}_{k,n}(t)=Pr\{K(t)=k,N(t)=n\}$. Also denote the sum of the modified degrees of all nodes in the network by *S*_*n*_. Then, when the network has size *n* − 1, one has ${S}_{n-1}=\sum _{i=1}^{n-1}{\hat{k}}_{i}^{(n-1)}$, thus ${S}_{n}=\sum _{i=1}^{n}{\hat{k}}_{i}^{(n)}=\sum _{\genfrac{}{}{0ex}{}{i=1}{i\ne {i}^{^{\prime} }}}^{n-1}{\hat{k}}_{i}^{(n-1)}+(\hat{{k}_{{i}^{^{\prime} }}^{(n-1)}+1})+{\hat{k}}_{n}^{(n)}$.

As can be verified by examining the state transition rate diagram in Fig. 4, the dynamics of the probability mass function satisfy the following equation:

4 $$\begin{array}{rcl}\frac{d}{dt}{p}_{k,n}(t) & = & \lambda (\widehat{k-1}){p}_{k-\mathrm{1,}n-1}(t)+\lambda (\sum _{i\ne \ast }{\hat{k}}_{i}){p}_{k,n-1}(t)-\lambda {S}_{n}{p}_{k,n}(t)\\  & = & \lambda (\widehat{k-1}){p}_{k-\mathrm{1,}n-1}(t)+\lambda ({S}_{n-1}-\hat{k}){p}_{k,n-1}(t)-\lambda {S}_{n}{p}_{k,n}(t\mathrm{).}\end{array}$$

for all *n*, but the last term of eq. (4) will vanish at the boundary $n={N}_{{\mathcal{T}}}$.

Summing eq. (4) over all the possible network size *n*, from initial network size *n*_0_ to ${N}_{{\mathcal{T}}}$, gives the dynamics of node *’s degree *k* (for $k\in \{\mathrm{0,}\cdots ,{N}_{{\mathcal{T}}}\}$) as follows (with detail steps illustrated in Sec. S3.1 in SI):

5 $$\frac{d}{dt}{p}_{k}(t)=\lambda (\widehat{k-1})({p}_{k-1}(t)-{p}_{k-\mathrm{1,}{N}_{{\mathcal{T}}}}(t))-\lambda \hat{k}({p}_{k}(t)-{p}_{k,{N}_{{\mathcal{T}}}}(t))\mathrm{.}$$

For simplicity of analysis, one could remove the small boundary terms $\lambda (\widehat{k-1}){p}_{k-\mathrm{1,}{N}_{{\mathcal{T}}}}(t)$ and $\lambda \hat{k}{p}_{k,{N}_{{\mathcal{T}}}}(t)$ (which vanishes as ${\mathcal{T}}\to \infty$), it will result in $\frac{d}{dt}{p}_{k}(t)=\lambda (\widehat{k-1}){p}_{k-1}(t)-\lambda \hat{k}{p}_{k}(t)$, i.e., eq. (2) for the case without burst structure *k* ^*b*^. Similar aggregation of state could be done for the case of MC model with bursty arrivals, leading to the eq. (2).

### Methods for Theoretical Results

The proofs of Theorem 1 and Theorem 2 are provided in Sec. S3.4 and S3.5 of SI respectively, which are similar to this one. This section focuses on Proof of Theorem 3 under the default MC model; the proof for the general case under the trichotomy structure (i.e., eq. (1)) is presented in Sec. S3.6 of SI. The proof for the most general form of network degree distribution for any form of modified degree (or attachment kernel) $\hat{k}$ is also given in Theorem 3 in Sec. S3.3 of SI.

Define the ${\mathcal{T}}$-truncated Laplace transforms of *p*_*k*_(*t*) and ${p}_{k,{N}_{{\mathcal{T}}}}(t)$ as ${P}_{k}(s)\triangleq {\int }_{0}^{{\mathcal{T}}}{e}^{-st}{p}_{k}(t)dt$ and ${P}_{k,{N}_{{\mathcal{T}}}}(s)\triangleq {\int }_{0}^{{\mathcal{T}}}{e}^{-st}{p}_{k,{N}_{{\mathcal{T}}}}(t)dt$.

By taking the ${\mathcal{T}}$-truncated Laplace transform on both sides of eq. (5) and using the integration-by-parts identity, i.e., ${\int }_{0}^{{\mathcal{T}}}{e}^{-st}\frac{d{p}_{k}(t)}{dt}dt=[{e}^{-st}{p}_{k}(t)]{|}_{t=0}^{t={\mathcal{T}}}+s{\int }_{0}^{{\mathcal{T}}}{e}^{-st}{p}_{k}(t)dt=-{p}_{k}\mathrm{(0)}+{e}^{-s{\mathcal{T}}}{p}_{k}({\mathcal{T}}\,)+s{P}_{k}(s)$, one can get, $\forall k\ge 1$,

6 $$\begin{array}{ccc}s{P}_{k}(s)-{p}_{k}(0)+{e}^{-s{\mathcal{T}}}{p}_{k}({\mathcal{T}}) & = & \lambda (\hat{k-1})({P}_{k-1}(s)-{P}_{k-1,{N}_{{\mathcal{T}}}}(s))-\lambda \hat{k}({P}_{k}(s)-{P}_{k,{N}_{{\mathcal{T}}}}(s))\\ \therefore {P}_{k}(s) & = & (\frac{\lambda (\hat{k-1})}{s+\lambda (\hat{k})}){P}_{k-1}(s)+\frac{{p}_{k}(0)+{B}_{k,{N}_{{\mathcal{T}}}}(s)-{e}^{-s{\mathcal{T}}}{p}_{k}({\mathcal{T}}\,)}{s+\lambda (\hat{k})}\\  & = & {\sum }_{i=1}^{k}({\prod }_{j=i}^{k-1}(\frac{\lambda \hat{j}}{s+\lambda (\hat{j+1})}))(\frac{{p}_{i}(0)+{B}_{i,{N}_{{\mathcal{T}}}}(s)-{e}^{-s{\mathcal{T}}}{p}_{i}({\mathcal{T}}\,)}{s+\lambda \hat{i}})\end{array}$$

where ${B}_{i,{N}_{{\mathcal{T}}}}(s)\triangleq \lambda (-(\widehat{k-1}){P}_{k-\mathrm{1,}{N}_{{\mathcal{T}}}}(s)+(\hat{k}){P}_{k,{N}_{{\mathcal{T}}}}(s))$ and ${e}^{-s{\mathcal{T}}}{p}_{i}({\mathcal{T}})$ are small for all *i* (and asymptotically converge to 0 as ${\mathcal{T}}\to \infty$) in typical settings of system parameters. Specifically, the upper bound ${\mathcal{U}}$ is typically non-trivial, i.e., ${\mathcal{U}}\ll {N}_{{\mathcal{T}}}$, so $\hat{i}$ is upper bounded, and thus this part of error $\lambda \hat{i}{P}_{i,{N}_{{\mathcal{T}}}}(s)$ vanishes as ${\mathcal{T}}\to \infty$.

Applying the eq. (6) to the trichotomy model (i.e., eq. (1)), one gets:

$${P}_{k}(s)=\{\begin{array}{cc}\frac{\mathop{{p}_{1}}\limits^{ \sim }}{\lambda L}{(\frac{\lambda L}{s+\lambda L})}^{k}, & \text{if}\,1 < k\le {\mathcal{L}}\\ \frac{\mathop{{p}_{1}}\limits^{ \sim }}{\lambda {\mathcal{L}}}\prod _{i={\mathcal{L}}}^{k-1}(\frac{\lambda (i)}{s+\lambda (i+1)}){(\frac{\lambda L}{s+\lambda L})}^{{\mathcal{L}}}, & \text{if}\,{\mathcal{L}} < k\le {\mathcal{U}}\\ \frac{{\mathcal{U}}\,\mathop{{p}_{1}}\limits^{ \sim }}{\lambda {\mathcal{L}}U}{(\frac{\lambda U}{s+\lambda U})}^{k-{\mathcal{U}}}\prod _{i={\mathcal{L}}}^{{\mathcal{U}}-1}(\frac{\lambda (i)}{s+\lambda (i+1)}){(\frac{\lambda L}{s+\lambda L})}^{{\mathcal{L}}}, & \text{if}\,{\mathcal{U}} < k\le {N}_{{\mathcal{T}}}\end{array}$$

It will be shown in Sec. S4 of SI that the residential-time of the node * has an exponential distribution with parameter *λγ* and a normalization constant $\frac{1}{1-{e}^{-\lambda \gamma {\mathcal{T}}}}$. After averaging *p*_*k*_(*t*) with this residential-time distribution, one gets

7 $${p}_{k}={E}_{T}[{p}_{k}(t)|N( {\mathcal L} )={N}_{ {\mathcal L} }]={\int }_{0}^{ {\mathcal L} }{p}_{k}(t)\frac{\lambda \gamma {e}^{-\lambda \gamma t}}{1-{e}^{-\lambda \gamma  {\mathcal L} }}dt=\frac{\lambda \gamma }{1-{e}^{-\lambda \gamma  {\mathcal L} }}{P}_{k}(\lambda \gamma )$$

which gives the result as stated in the theorem since $\frac{\lambda \gamma }{\lambda  {\mathcal L} }\prod _{i= {\mathcal L} }^{k-1}(\frac{\lambda (i)}{\lambda \gamma +\lambda (i+1)}){(\frac{\lambda L}{\lambda \gamma +\lambda L})}^{ {\mathcal L} }=\frac{\gamma }{ {\mathcal L} }\frac{\frac{(k-1)!}{( {\mathcal L} -1)!}}{\frac{{\rm{\Gamma }}(k+\gamma +1)}{{\rm{\Gamma }}(\gamma + {\mathcal L} +1)}}{(\frac{L}{\gamma +L})}^{ {\mathcal L} }$, which is a power-law with exponent −(*γ* + 1).

### Methods for Experimental Results

For fitting the default MC model (or generally with fix mixing probability), a maximum likelihood procedure, generalizing existing literature^19^ to the case with “trichotomy” is proposed in Sec. S9 of SI. For the fitting with the general MC model with mixing probability, one could use expectation-maximization (EM) algorithm, by refering the proposed procedure for the default MC model (with fixed mixing probability) as the maximization step in EM, and update the mixing probability using an expectation step as in that of exponential random graph model with mixture^20, 21^. In Sec. S9, another fast heuristic method (without the need of this alternating procedure (EM)) is introduced to handle the case with small numbers of mixing probabilities.

## Electronic supplementary material

Supplementary Information
