# Publisher Correction: A Unified Framework for Complex Networks with Degree Trichotomy Based on Markov Chains

**DOI:** 10.1038/s41598-019-56285-2

**Published:** 2019-12-19

**Authors:** David Shui Wing Hui, Yi-Chao Chen, Gong Zhang, Weijie Wu, Guanrong Chen, John C. S. Lui, Yingtao Li

**Affiliations:** 1 Huawei Technologies Co. Ltd., Hong Kong, China; 2 City University of Hong Kong, Hong Kong, China; 3 The Chinese University of Hong Kong, Hong Kong, China; 4 Huawei Technologies Co. Ltd., Shenzhen, China

Correction to: *Scientific Reports* 10.1038/s41598-017-03613-z, published online 16 June 2017

This Article contains typographical errors in the Results section under the subheading “Theorem 3”, where

$$({c}_{2i},{k}_{2i}^{s})=\{\begin{array}{c}(p {\mathcal L} i\cdot \frac{L}{\gamma }, {\mathcal L} +1)\\ (c,i)\end{array}$$

should read:

$$({c}_{2i},{k}_{2i}^{s})=\{\begin{array}{c}({p}_{ {\mathcal L} i}\cdot \frac{L}{\gamma }, {\mathcal L} +1)\\ (c,i)\end{array}$$

and

$$({c}_{3i},{k}_{3i}^{s})=\{\begin{array}{c}(pUi\cdot \frac{U}{\gamma },U+1)\\ (c,i)\end{array}$$

should read:

$$({c}_{3i},{k}_{3i}^{s})=\{\begin{array}{c}({p}_{{\mathscr{U}}i}\cdot \frac{{\mathscr{U}}}{\gamma },{\mathscr{U}}+1)\\ (c,i)\end{array}$$

