# Supplementary material for: A Unified Framework for Complex Networks with Degree Trichotomy Based on Markov Chains
Source: Sci Rep. 2017 Jun 16;7:3723. doi: 10.1038/s41598-017-03613-z (PMC5473852; doi:10.1038/s41598-017-03613-z)
Supplement: Supplementary file 1 — Supplementary Information [file 41598_2017_3613_MOESM1_ESM.pdf]

Supplementary Information for “A Unified Framework for Complex Networks with Degree Trichotomy Based on Markov Chains”

David Shui Wing Hui[1], Yi-Chao Chen[1], Gong Zhang[1], Weijie Wu[1], Guanrong Chen[2], John C. S. Lui[3], and Yingtao Li[1]

[1]Huawei Technologies Co. Ltd., China
[2]City University of Hong Kong
[3]The Chinese University of Hong Kong

ABSTRACT

This supplementary information document provides further explanations of the proposed model, particularly to those statements that were only mentioned briefly in the main text, detailed proofs of theorems, the data fitting method, as well as further experimental examples.

S1 Introduction

Recall from the main text of the paper, the MC model is proposed to represent the evolution processes in complex network. Under this model, each node in the network starts with a (possibly) random starting degree and evolves according to three phases of development — initializing phase, fast-evolving phase and maturing phase. In retrospect, the fast-evolving phenomenon in different networks is also reported in existing models, such as the BA model and its variants mentioned in the introduction section of the main text. The major differences of this proposed MC model, as compared to existing models, are on the initializing phase and the maturing phase, both representing physical constraints but in different contexts - the initializing phase for characterizing the constraint due to system set-up time, and the maturing phase for characterizing the constraint due to physical, economical and technological limitations. These differences are the major causes of the observed trichotomy in density functions of various networks, particularly next generation information networks. Furthermore, differing from the existing literature, the proposed model includes a burst attractive point (which can model the unaddressed occasionally simultaneous connections reported in many complex networks in the literature), and allows random starting capabilities (which can provide further flexibility in modeling network initialization). In particular, it helps in providing a more reasonable final close-form expression in node-degree distribution of the network presented in Theorem 3 of the main text, which is closer to the empirical results, as well as the resultant model is more close to real situations. Specifically, in the bursty arrival phase, each time when a new information consumption attempt arrives, it will connect to one or multiple existing attempts at random. Such a possibility of multiple connections (also called burst attractiveness of a node) is also needed in the model as several important networks also have a bursty change (instead of incremental change) at certain special instance. Example networks include citation networks and and online social networks are discussed in the main text. Further example also include the next generation vehicular network: when a new vehicle comes into a communication zone, it will be in contact (or in feasible communication region) with multiple (instead of one) vehicles. In the proposed MC model, when a node reaches burst attractive point k^b, the probability of making $k-k^b$ further number of connections simultaneously in the next arrival event to this node is modeled by a network-specific parameter $p_{k^b \to k}$. A more technical comparison between the MC model and existing models are given as follows.

Comparison with existing models

There has been several existing models on network formation mechanism to account for the empirically observation in individual parts of degree trichotomy in degree distribution (in log-log plot)[1] – power-law behavior (i.e., linearity in medium degree), high-degree cutoff[2] and small-degree flattening[3]. There are several major differences between the proposed MC model and the existing models. First, the MC model provides the first unifying model that can capture all features in degree trichotomy, while existing models can only cover part of the degree trichotomy, even though this phenomenon is empirically repeatedly reported in existing literature. Second, the methodologies used in MC model is a stochastic model, while existing models mainly based on continuum theory, i.e., the stochastic behavior of node degree dynamics is approximated by a continuum (i.e., deterministic)

dynamics. In continuum theory, degree is deterministically growing over time and the network has nodes arriving with fixed interarrival time. There are clear deviations of the continuum theory from the realistic environment, which is random as the attachment process (i.e., new edge formation) is a random choice in every complex networks. One would expect that different assumptions on the random environment would lead to different network characteristic, while continuum theory would fail to characteristic those assumptions explicitly due to its deterministic approximation nature. In the MC model, the choice of the random environment is explicitly specified as Markovian, specifically the network size has independent increment, and a new node's arrival rate only depends on the current number of nodes in the network. Third, the closed-form formula for the general case could be given under the MC model which are not present in existing works. There are also other effects, including the effect of various distributions of starting degrees and burst structure, which could only be accounted in a stochastic model, are thus absent in existing models, but are provided explicitly in our proposed MC model. For ease of references, a list of existing model for accounting individual effects of degree trichotomy, in our terminologies, are given as follows:

Power-law behavior: Preferential attachment is proposed in BA model. Using our terminologies, the attachment kernel, denoted as $\hat{k}$ is proportional to the degree k, is given by:

$$\hat{k} = c \cdot k \tag{1}$$

where c is a proportionally constant. Continuum theory showed a one-phase power-law distribution is resulted under this model.

High-degree cutoff: Sublinear preferential attachment[2,4] is used, i.e., the attachment kernel is given by:

$$\hat{k} = c \cdot k^{\nu} \tag{2}$$

where $\nu < 1$ is a constant used to model sublinear effect in high degree and c is the proportionality constant. Continuum theory[2] results in (approximate) degree distribution with two power-law regimes (with second regime act as a cut-off).

Small-degree flattening: Initial attractiveness factor is used (in generic network[5], in finite-size networks[6], and with link addition, rewiring, and node addition consideration[7]), i.e., the attachment kernel is given by:

$$\hat{k} = c(A + k) \tag{3}$$

where $A > 0$ is an initial attractiveness constant and c is the proportionality constant. Continuum theory[5] results in a degree distribution with small-degree flattening (SDF) (i.e., degree distribution lower than power law prediction).

From the existing literature, i.e., equations (1), (2) and (3), it is not easy to find out a direct generalization that can produce degree trichotomy while analytically tractable.

The proposed MC model , in contrast, to be elaborated in this SI, can provide a closed-form formula on network degree distribution for the general attachment kernel $\hat{k}$ in theorem 3, and in particular, it reduced to theorem 9 for the case of interest – the attachment kernel exhibiting trichotomy, i.e.,

$$\hat{k}_i = \begin{cases} L, & \text{if } 0 \le k_i \le \mathscr{L} \\ k_i, & \text{if } \mathscr{L} < k_i \le \mathscr{U} \\ U, & \text{if } \mathscr{U} < k_i \le N_{\mathscr{T}}, \end{cases} \tag{4}$$

Several auxiliary effects are also discussed in the existing models to model other realistic features of network generation, and they could also be incoporated in the MC model as follows. (1) To account for heterogeneity of node degree dynamics, fitness model[8–11] is proposed where node i has individual fitness values η_i which changes its attachment probability from proportional to degree k_i to $\hat{k}_i = c(\eta_i k_i)$. Closed-form degree distribution may only be achieved under specific scenarios[8], or asymptotic regime[11]. Its major disadvantage is that there are many model parameters, easily leading to over-fitting problem. (2) To account for the effect of node deletion[12] and internal links[13], a presumed form with phase transition (i.e., a power-law with exponential cut-off) is assumed, as node deletion can also give cut-off[14] in degree distribution. But these effects are not applicable to citation network, as citation count always increases. Since degree trichotomy is also observed empirically without those auxiliary effects (i.e., heterogeneity of degree dynamics and node deletions)[15], the MC model is proposed as a mechanism to give rise to the trichotomy feature first. One could add those effects to our trichotomy result using continumm theory, by changing the external arrival rate λ to account further those internal links formation (by increasing λ) and node deletion (by reducing λ), and using our general result for MC model for the case with fitness. For including the auxiliary effects into the proposed MC model, without using any approximation like the continuum theory (so as to account the stochastic effect appropriately), or include other effects like similarity[16] and causality[17], substantial analysis still needed, which is left as future work.

Stochastic evolution modeling: To our best knowledge, most existing works rely on continuum (deterministic) theory, the state-of-art result on stochastic model in generating complex network is mainly Yule process[18,19], which is a birth process with a rate proportional to the state variable. And it can be viewed as a BA model after proper scaling of the interarrival time. Fellow researchers choose to use continuum theory, instead of generalizing Yule process, likely because a deterministic model is more easier to analyze than its stochastic counterpart, despite its deficiency mentioned above. The proposed general MC model can be considered as a generalization of Yule process, to a more general Markov process with a rate accounting both the state variable and the node degree bounds on the connection probabilities due to physical constraints, and a bursty arrival (specifically, it differs from the Yule process[18,19] by having a lower bound and an upper bound on the birth rates, modified with a bursty arrival. For example, as shown in Figure 1 in the main text, at the burst location $k^b = 0$, $p_{0\to 1}, p_{0\to 2}, \ldots, p_{0\to \mathscr{L}+1}$ are the probabilities of having burst size of $1, 2, \ldots, \mathscr{L}+1$ connections due to the arrival of new nodes respectively, which is a structure absent in Yule process). Yule process is a special case of MC model, as it has only one phase in the MC model — the fast-evolving phase, corresponding to the preferential attachment mechanism based only on the degrees of the existing nodes but not related to the size or other physical constraints of the current network structure. This generalization is important, as in Experiment Section below, it will be shown that there are some statistical evidence that most real-world networks have nodes that are well-modeled by the initializing and maturing phases of the present model.

In short, related works on modeling the realistic effects either the high-degree cutoff or small-degree saturation are difficult to be put in a unified framework for a closed-form solution analysis. Furthermore, some practical issues like varieties in starting degree and burst structure are not discussed in existing literature, since it is more naturally incorporated in a stochastic model, but most existing works rely on continuum (deterministic) theory. Therefore, the proposed MC model, as a more general modeling framework, could possibly be a more realistic choice than existing models in representing the generation processes of a general complex networks.

S2 Table of Notations

For the ease of reference, a list of important system parameters used in this Supplementary Information document (and the main text) is summarized in Table 1.

As time t increases from time 0 to the observation time $\mathscr{T}$, the system parameters are fixed, but the system variables (which are defined in Table 2) could change. In particular, the main text defined an important system variable — the modified degree $\widehat{k_i}$ in equation (4), based on the above system parameters. Recall in the main text, this $\hat{k}_i$ plays an essential role in the network formation process as it is the proportionality constant of the attachment probability of a new node to a particular existing node i. This Supplementary Information document will further illustrate its important role in changing the system dynamics and eventually the node-degree distribution in the following Section.

Table 1. A list of important system parameters

Notation	Meaning
$\mathscr{L}$	Lower boundary value of the degree of a node signifying the end of the initializing phase
$\mathscr{U}$	Upper boundary value of the degree of a node signifying the start of the maturing phase
L	Lower bound of modified degree for lower bounding the attachment probability in the whole initializing phase
U	Upper bound of modified degree for upper bounding the attachment probability in the whole maturing phase
λ	Normalized arrival rate, i.e., average arrival rate of new attempts divided by L
k^b	Burst attractive degree, i.e., a special degree where burst arrival to a node could happen (optional)
$p_{k^b\to k}$	A parameter to control the probability of burst size being $k-k^b$ at burst attractive degree (optional)
$p_i(0)$	The probability of the starting degree of a node is i
$\mathscr{T}$	Current observation time of the system
$N_{\mathscr{T}}$	The number of nodes in the system at time $\mathscr{T}$

It is remarked that $\mathscr{L}$, $\mathscr{U}$, L is the minimum set of parameters to model degree trichotomy. Specifically one can use the default MC model with $U = \mathscr{U}$, it is still possible to model degree trichotomy.

Also for convenience, a list of important system variables used in this paper is also summarized in Table 2.

In later discussions, the subscript * used in the notations of the above variables (presented in Table 2) would be omitted, whenever the specified node * is explicitly defined in the text, e.g., k will be used as an abbreviation of k_*, and T will be used as an abbreviation of T_*. Since the values of the system variables depend on time t, this time variable t will be explicitly specified in the text whenever there is a need of clarification, e.g., $p_{k,n}(t)$ is used to denote the probability that "the degree of the specified node $* = k$, and the network size $= n$, at time t".

Table 2. A list of important system variables

Notation	Meaning
k_*	Degree of a specific node *
n	Network size - i.e., number of nodes in the network
$\hat{k_*}$	Modified degree of a specific node * defined in equation (4)
T_*	Residential-time of a specific node *, i.e., $\mathscr{T} - t_*$, where t_* is the arrival time of node *
t	Running time index of the Markov Chains (M.C.)

S3 Proofs of Theorems in the Methodology Section

S3.1 Relationship between different dynamics equations in the MC model

In the section, we explain one could arrive at the degree dynamics of an individual node in the complex network (shown in Figure 1 of the main text), from the dynamics of the full state variables in the MC model, as mentioned in the methodology section of the main text. For illustrating the derivation steps, it suffices to consider the case of the MC model without bursty arrival structure. Recall the two key dynamic equations in the main text under this case:

- **Full System State Dynamics.** The dynamics of the probability mass function of the state variable — the degree k of a specific node * (for $k \in \{0, \cdots N_{\mathscr{T}}\}$) and the network of size n (for $n \in \{1, \cdots N_{\mathscr{T}}\}$) satisfy the following equation:

  For $n \in \{1, \cdots N_{\mathscr{T}} - 1\}$**:**

$$\begin{aligned}\frac{d}{dt} p_{k,n}(t) &= \lambda \left(\widehat{k-1}\right) p_{k-1,n-1}(t) + \lambda \left(\sum_{i \neq *} \hat{k}_i\right) p_{k,n-1}(t) - \lambda S_n p_{k,n}(t) \\ &= \lambda \left(\widehat{k-1}\right) p_{k-1,n-1}(t) + \lambda (S_{n-1} - \hat{k}) p_{k,n-1}(t) - \lambda S_n p_{k,n}(t)\end{aligned} \tag{5}$$

  For $n = N_{\mathscr{T}}$**:**

$$\frac{d}{dt} p_{k,n}(t) = \lambda \left(\widehat{k-1}\right) p_{k-1,n-1}(t) + \lambda \left(S_{n-1} - \hat{k}\right) p_{k,n-1}(t) \tag{6}$$

  with the boundary conditions $p_{k,n_*+k-2}(t) = 0$ for all $k \geq 0$ (assuming that when node * arrives, the network contains n_* of nodes) and $p_{k,N_{\mathscr{T}}+d}(t) = 0$ for all $k \geq 0$ and $d \geq 1$.

  This dynamic equation means that as a new node arrives (i.e., n increases by 1), either the node under consideration can attract the new node (i.e., k increases by 1) or it cannot (i.e. k remain the same).

  A remark for the case with burst structure is that there could be some transition probability from multiple new node arrive in a dependent manner (instead of one by one), in such case degree k can also increases by more than 1.

- **Individual Degree Dynamics.** The dynamics of the degree k (for $k \in \{0, \cdots N_{\mathscr{T}}\}$) of a specified node * satisfy

$$\frac{d}{dt} p_k(t) = \lambda \left(\widehat{k-1}\right) \left(p_{k-1}(t) - p_{k-1,N_{\mathscr{T}}}(t)\right) - \lambda \left(\hat{k}\right) \left(p_k(t) - p_{k,N_{\mathscr{T}}}(t)\right) \tag{7}$$

  and after removing the small boundary terms $\lambda(\widehat{k-1}) p_{k-1,N_{\mathscr{T}}}(t)$ and $\lambda \hat{k} p_{k,N_{\mathscr{T}}}(t)$ (which vanishes as $\mathscr{T} \to \infty$), the degree dynamics satisfy (as shown in the main text)

$$\frac{d}{dt} p_k(t) = \lambda \left(\widehat{k-1}\right) p_{k-1}(t) - \lambda \left(\hat{k}\right) p_k(t). \tag{8}$$

  which can capture the degree dynamics of many existing and other interesting models.

Example: Under Degree-Trichotomy model, equation (8) becomes:

$$\frac{d}{dt}p_k(t) = \begin{cases} -\lambda L p_k(t), & \text{if } k = k^s \\ \lambda L p_{k-1}(t) - \lambda L p_k(t), & \text{if } k^s < k \leq \mathscr{L} \\ \lambda (L) p_{k-1}(t) - \lambda k p_k(t), & \text{if } k = \mathscr{L}+1 \\ \lambda (k-1) p_{k-1}(t) - \lambda k p_k(t), & \text{if } \mathscr{L}+1 < k \leq \mathscr{U} \\ \lambda (k-1) p_{k-1}(t) - \lambda U p_k(t), & \text{if } k = \mathscr{U}+1 \\ \lambda U p_{k-1}(t) - \lambda U p_k(t), & \text{if } \mathscr{U}+1 < k \leq N_{\mathscr{T}} \end{cases} \tag{9}$$

where k^s is the starting degree of the node. Noted that equation (9) could be further simplified if $L = \mathscr{L}$ or $U = \mathscr{U}$.

In fact, **these two dynamics are related by a simple summation** as follows: equation (7) is obtained by summing equation (5) over n from 2 to $N_{\mathscr{T}}$ which gives

$$\begin{aligned} \frac{d}{dt}\sum_{n=2}^{N_{\mathscr{T}}} p_{k,n}(t) = \lambda \left(\widehat{k-1}\right) \left(\sum_{n=2}^{N_{\mathscr{T}}} p_{k-1,n-1}(t) + p_{k-1,N_{\mathscr{T}}}(t) - p_{k-1,N_{\mathscr{T}}}(t) \right) \\ + \lambda \sum_{n=2}^{N_{\mathscr{T}}} \left(S_{n-1} - \hat{k}\right) p_{k,n-1}(t) - \lambda \hat{k} p_{k,N_{\mathscr{T}}}(t) + \lambda \hat{k} p_{k,N_{\mathscr{T}}}(t) - \lambda \sum_{n=2}^{N_{\mathscr{T}}-1} S_n p_{k,n}(t). \end{aligned} \tag{10}$$

Rewrite equation (10) with the definition of the density function of the degree k given by $p_k(t) = \sum_{n=2}^{N_{\mathscr{T}}} p_{k,n}(t)$, equation (7) is thus obtained. Similar aggregation of state (i.e. summation of probability) could be done for the case of MC model with bursty arrival, and resulting in a general degree dynamics, depicted in Figure 1 of the main text, as follows:

$$\frac{d}{dt}p_k(t) = \begin{cases} -\lambda \left(\hat{k}\right) p_k(t) + b_{k,N_{\mathscr{T}}}(t), & \text{if } k = 0 \\ \lambda \left(\widehat{k-1}\right) p_{k-1}(t) - \lambda \left(\hat{k}\right) p_k(t) + b_{k,N_{\mathscr{T}}}(t), & \text{if } 1 \leq k \leq k^b \\ \lambda \left(\widehat{k^b}\right) p_{k^b \to k} P_{k^b}(t) - \lambda \left(\hat{k}\right) p_k(t) + b_{k,N_{\mathscr{T}}}(t), & \text{if } k = k^b + 1 \\ \lambda \left(\widehat{k-1}\right) p_{k-1}(t) + \lambda \left(\widehat{k^b}\right) p_{k^b \to k} p_{k^b}(t) - \lambda \left(\hat{k}\right) p_k(t) + b_{k,N_{\mathscr{T}}}(t), & \text{if } k \geq k^b + 2 \end{cases} \tag{11}$$

$$= \lambda \left(\widehat{k-1}\right) p_{k-1}(t) \mathbb{1}\left(k \neq \left\{0, k^b+1\right\}\right) + \lambda \left(\widehat{k^b}\right) p_k^0 p_{k^b}(t) \mathbb{1}\left(k \geq k^b + 1\right) - \lambda \left(\hat{k}\right) p_k(t) + b_{k,N_{\mathscr{T}}}(t), \tag{12}$$

where $b_{k,N_{\mathscr{T}}}(t) \triangleq \lambda \left(-\left(\widehat{k-1}\right) p_{k-1,N_{\mathscr{T}}}(t) \mathbb{1}(k \neq 0) + \left(\hat{k}\right) p_{k,N_{\mathscr{T}}}(t) \right)$.

It is further noted that when deriving equation (8), two boundary terms $\lambda(\widehat{k-1}) p_{k-1,N_{\mathscr{T}}}(t)$, and $\lambda \hat{k} p_{k,N_{\mathscr{T}}}(t)$ in (7) (i.e., $b_{k,N_{\mathscr{T}}}(t)$ are omitted, as they are very small as compared to other terms in equation (7). Actually, in $p_k(t) = \sum_{n=2}^{N_{\mathscr{T}}} p_{k,n}(t)$, the term $p_{k,N_{\mathscr{T}}}(t)$ is the smallest term in the sum, and it vanishes as $\mathscr{T} \to \infty$. Hence, neglecting these boundary terms would not cause significant difference in equation (8), but can greatly simplify the subsequent analysis.

Using the above dynamic equations, with a particular setting of the system parameters L, $\mathscr{L}$, $\mathscr{U}$, U, some results of the classical models, including Poisson network model, Exponential network model, and Power-law network model, can be reformulated in the proposed MC model as stated in the theorems of the main text. Furthermore, the proposed MC model can derive new closed-form results on degree distributions which fit much better with the empirical data than the classical models. In particular, this MC model is the first model to explain the observed trichotomy phenomenon. All the aforementioned results are proved in the remaining part of this section. Proof of statements of the Poisson and Exponential network model is presented in subsection S3.4. Proof of statements of power-law network model is presented in subsection S3.5. Furthermore, detail proof of statements of the general case (as a supplement to the proof in the main text) is presented in subsection S3.5.

S3.2 A simple lemma on difference equation

Lemma 1 *Consider a k-parameterized difference equation in $P_k(s)$ of the following form (throughout this paper, $P_k(s)$ is the Laplace transform (as a function in s) of degree distribution of a node $p_k(t)$ over time t):*

$$P_k(s) = Q_{k-1}(s) P_{k-1}(s) + R_k(s), \qquad \forall k \geq k^s + 1 \tag{13}$$

where k and k^s are nonnegative integers, and $Q_k(s)$ and $R_k(s)$ are some given functions in s for all $k \geq k^s$, where k^s is some arbitrary starting degree.

Suppose the initial conditions are $P_{k^s}(s) = R_{k^s}(s)$, it has the following solution

$$P_k(s) = \sum_{i=k^s}^{k-1} \left(Q_{i\to k-1}(s) R_i(s)\right) + R_k(s), \qquad \forall k \geq k^s + 1 \tag{14}$$

where $Q_{i\to k-1}(s) \triangleq \prod_{j=i}^{k-1} Q_j(s)$, for all $k-1 \geq i \geq k^s$.

Proof It can be easily proved by mathematical induction as follows. Let the equation (14) to be the statement $S(k)$ for induction test. By equation (13), $S(k^s+1)$ is true as $Q_{k^s\to k^s}(s) = Q_{k^s}(s)$. Suppose $S(k)$ is true for $k \geq k^s+1$, by equation (13), and then plug in equation (14) to $P_k(s)$, then $P_{k+1}(s)$ can be expressed in the same form as equation (14), as illustrated as follows:

$$\begin{aligned} P_{k+1}(s) &= Q_k(s) P_k(s) + R_{k+1}(s) \\ &= Q_k(s) \left(\sum_{i=k^s}^{k-1} \left(\left(\prod_{j=i}^{k-1} Q_j(s) \right) R_i(s) \right) + R_k(s) \right) + R_{k+1}(s) \\ &= \sum_{i=k^s}^{k} \left(\left(\prod_{j=i}^{k} Q_j(s) \right) R_i(s) \right) + R_{k+1}(s) \end{aligned} \tag{15}$$

thus $S(k+1)$ is true. ∎

S3.3 General solution methodology in obtaining degree distribution under the MC model

Step 1: Laplace Transform of degree distribution

Define the $\mathscr{T}$-truncated Laplace transform of any function of t, $f(t)$, as follows:

$$F(s) \triangleq \int_0^{\mathscr{T}} e^{-st} f(t) dt. \tag{16}$$

In particular, the $\mathscr{T}$-truncated Laplace transform of $p_k(t)$ is denoted as $P_k(s) = \int_0^{\mathscr{T}} e^{-st} p_k(t) dt$.

There is a useful lemma on $P_k(s)$ written as follows:

Lemma 2 *The Laplace transform of the degree distribution $p_k(t)$ under the MC model is*

$$P_k(s) = \sum_{i=0}^{k} q_i(s) \frac{1}{\lambda \hat{k} \left(1 + (p^0_{k+1} - 1)\, \mathbb{1}(k = k^b)\right)} \prod_{j=j}^{k} \frac{\lambda \hat{j}}{s + \lambda \hat{j}} \left(1 + (p^0_{j+1} - 1)\, \mathbb{1}\left(j = k^b\right)\right) \tag{17}$$

where

$$q_i(s) = p_k(0) - e^{-s\mathscr{T}} p_k(\mathscr{T}) + \lambda \left(\widehat{k^b}\right) p^0_k P_{k^b}(s)\, \mathbb{1}\left(k \geq k^b + 2\right) + B_{k,N_{\mathscr{T}}}(s) \tag{18}$$

Proof Using the rule of integration-by-parts, one has

$$\begin{aligned} \int_0^{\mathscr{T}} e^{-st} \frac{dp_k(t)}{dt} dt &= [e^{-st} p_k(t)]|_{t=0}^{t=\mathscr{T}} + s \int_0^{\mathscr{T}} e^{-st} p_k(t) dt \\ &= -p_k(0) + e^{-s\mathscr{T}} p_k(\mathscr{T}) + s \int_0^{\mathscr{T}} e^{-st} p_k(t) dt. \end{aligned} \tag{19}$$

Therefore, by taking the $\mathscr{T}$-truncated Laplace transform on degree dynamics of the MC model (i.e. the differential-difference equation (12)), one has, for $k \geq 0$

$$sP_k(s) - p_k(0) + e^{-s\mathscr{T}} p_k(\mathscr{T}) = \begin{cases} -\lambda\left(\hat{k}\right) P_k(s) + B_{k,N_{\mathscr{T}}}(s), & \text{if } k = 0 \\ \lambda\left(\widehat{k-1}\right) P_{k-1}(s) - \lambda\left(\hat{k}\right) P_k(s) + B_{k,N_{\mathscr{T}}}(s), & \text{if } 1 \leq k \leq k^b \\ \lambda\left(\widehat{k^b}\right) p^0_k P_{k^b}(s) - \lambda\left(\hat{k}\right) P_k(s) + B_{k,N_{\mathscr{T}}}(s), & \text{if } k = k^b + 1 \\ \lambda\left(\widehat{k-1}\right) P_{k-1}(s) + \lambda\left(\widehat{k^b}\right) p^0_k P_{k^b}(s) - \lambda\left(\hat{k}\right) P_k(s) + B_{k,N_{\mathscr{T}}}(s), & \text{if } k \geq k^b + 2 \end{cases} \tag{20}$$

$$= \lambda\left(\widehat{k-1}\right) P_{k-1}(s)\, \mathbb{1}\left(k \neq \left\{0, k^b+1\right\}\right) + \lambda\left(\widehat{k^b}\right) p^0_k P_{k^b}(s)\, \mathbb{1}\left(k \geq k^b + 1\right) - \lambda\left(\hat{k}\right) P_k(s) + B_{k,N_{\mathscr{T}}}(s), \tag{21}$$

where $B_{k,N_{\mathscr{T}}}(s) \triangleq \lambda\left(-\left(\widehat{k-1}\right)P_{k-1,N_{\mathscr{T}}}(s)\mathbb{1}(k\neq 0)+\left(\hat{k}\right)P_{k,N_{\mathscr{T}}}(s)\right)$, $P_{k,N_{\mathscr{T}}}(s)$ is defined as the $\mathscr{T}$-truncated Laplace transform of $p_{k,N_{\mathscr{T}}}(t)$, i.e. $P_{k,N_{\mathscr{T}}}(s) \triangleq \int_0^{\mathscr{T}} e^{-st}p_{k,N_{\mathscr{T}}}(t)dt$, which is small for all k since $p_{k,N_{\mathscr{T}}}(t)$ is small for the reason mentioned above.

Thus, for $k \geq 1$,

$$
\begin{aligned}
P_k(s) = &\left(\frac{\lambda\left(\widehat{k-1}\right)}{s+\lambda\widehat{k}}\left(1+\left(p_{k^b\to k}-1\right)\mathbb{1}\left(k=k^b+1\right)\right)\right)P_{k-1}(s)\\
&+\left(\frac{\lambda\left(\widehat{k^b}\right)p_{k^b\to k}P_{k^b}(s)\mathbb{1}\left(k\geq k^b+2\right)+p_k(0)-e^{-s\mathscr{T}}p_k(\mathscr{T})+B_{k,N_{\mathscr{T}}}(s)}{s+\lambda\widehat{k}}\right),
\end{aligned}
\tag{22}
$$

with the initial condition

$$
P_0(s)=\frac{p_0(0)-e^{-s\mathscr{T}}p_0(\mathscr{T})+B_{0,N_{\mathscr{T}}}(s)}{s+\lambda\widehat{0}}. \tag{23}
$$

By treating equation (22) with initial condition (23) as a special case of equation (13), and then apply lemma 1, it results in the following:

The Laplace transform of the degree distribution $p_k(t)$ under the MC model is

$$
P_k(s)=\sum_{i=0}^{k-1}\left(Q^{MC}_{i\to k-1}(s)R^{MC}_i(s)\right)+R^{MC}_k(s), \qquad \forall k\geq 1 \tag{24}
$$

with initial condition

$$
P_0(s)=R^{MC}_0(s) \tag{25}
$$

where

$$
\begin{aligned}
&Q^{MC}_{i\to k-1}(s)\triangleq\prod_{j=i}^{k-1}Q^{MC}_j(s), \forall k-1\geq i\geq 0, \quad \text{in which } Q^{MC}_j(s)=\frac{\lambda\left(\widehat{j}\right)}{s+\lambda\left(\widehat{j+1}\right)}\left(1+\left(p^0_{j+1}-1\right)\mathbb{1}\left(j=k^b\right)\right)\\
&R^{MC}_k(s)=\frac{p_k(0)-e^{-s\mathscr{T}}p_k(\mathscr{T})+\lambda\left(\widehat{k^b}\right)p^0_kP_{k^b}(s)\mathbb{1}\left(k\geq k^b+2\right)+B_{k,N_{\mathscr{T}}}(s)}{s+\lambda\widehat{k}}, \forall k\geq 0.
\end{aligned}
\tag{26}
$$

After simplification of the above expression, the lemma as stated could be obtained. ∎

Step 2: Residential-time Distribution

Note that taking inverse Laplace transform of the above $P_k(s)$, would give $p_k(t)$, which only represents the degree distribution of one particular node * at time t if it arrives at time 0. More generally, if a node i arrives at time a_i, its degree distribution at the observation time $\mathscr{T}$ is given by $p_k(\mathscr{T}-a_i)$. Denote the residential-time of each node i by $T_i=\mathscr{T}-a_i$.

The degree distribution of the network is thus given by $p_k=\frac{1}{N_{\mathscr{T}}}\sum_{i=1}^{N_{\mathscr{T}}}\int_0^{\mathscr{T}}p_{k_i}(t)f_{T_i}(t)dt=\mathbb{E}_{i\sim U\{1,\cdots,N_{\mathscr{T}}\}}\mathbb{E}_{T_i}\left[p_{k_i}(t)\right]$, where $N_{\mathscr{T}}$ denotes the total number of nodes in the network at the observation time $\mathscr{T}$, and $f_{T_i}(t)$ denotes the probability density function of the residential-time T_i of node i. The symbol $i\sim U\{1,\cdots,N_{\mathscr{T}}\}$ represents that a node i is uniformly selected among the nodes ranged from 1 to $N_{\mathscr{T}}$.

In other words, the degree distribution of the network is given by $p_k=\mathbb{E}_T\left[p_k(t)\right]$, where T is the random variable denoting the residential-time of a randomly picked node, and $p_k(t)$ is the degree distribution of this randomly picked node with residential-time $T=t$. Thereafter, a crucial step of computing p_k is to find the distribution of the residential-time T.

The details of the calculation of the distribution of Residential-time T is presented in Section S4.

Step 3: Degree Distribution

Method 1: Using inverse Laplace Transform

When $\mathscr{T}$ is large, from the $\mathscr{T}$-Truncated Laplace Transform $P_k(s)$, one could obtain $p_k(t)$ by inverse Laplace Transform on $P_k(s)$, and compute the expectation over the residential time to get the degree distribution $p_k=\mathbb{E}_T\left[p_k(t)\right]$. This method is

particularly useful when expectation is easy to calculate. In particular, this method is used for accounting the degree distribution of a specific set of nodes in this paper where the residential time is a constant.

Method 2: Using the MC model's properties

This method is useful for accounting the degree distribution of all nodes in the network where the node dynamics are driven by the MC model.

From Section S4, at the observation time $\mathscr{T}$, the residential time distribution of the nodes under the dynamics driven by the MC model would be exponential distributed with some parameters $\lambda\gamma$, abbreviated as $exp(\lambda\gamma)$ (in which γ, will be called the residential-time parameter, and is determined by the system parameters of the MC model), i.e., the probability density function of the residential-time T, $f_T(t)$, is given by:

$$f_T(t) = \frac{\lambda\gamma e^{-\lambda\gamma t}}{1-e^{-\lambda\gamma\mathscr{T}}}, \text{ for } t \in [0, \mathscr{T}]. \tag{27}$$

Thus, the degree distribution of the network is given by

$$\begin{aligned} p_k &= \mathbb{E}_T[p_k(t)] \\ &= \int_0^{\mathscr{T}} p_k(t) f_T(t)\,dt \\ &= \frac{\lambda\gamma}{1-e^{-\lambda\gamma\mathscr{T}}}\int_0^{\mathscr{T}} p_k(t) e^{-\lambda\gamma t} dt \\ &= c_{\lambda\gamma}\lambda\gamma P_k(\lambda\gamma) \text{ where } c_{\lambda\gamma} \triangleq \frac{1}{1-e^{-\lambda\gamma\mathscr{T}}} \text{ is some normalization constant} \end{aligned} \tag{28}$$

After simplifying corollary 2, it results in the following useful lemma throughout the text of this Supplementary Information:

Theorem 3 *The the degree distribution of the network, p_k, under the MC model (with residential-time parameter γ) is*

$$p_k = \sum_{i=0}^{k} q_i p^s_{ki} d_{ki}, \qquad \forall k \geq 0 \tag{29}$$

where

$$q_i = p_i(0) + \frac{\widehat{k^b}}{c_{\lambda\gamma}\gamma} p_{k^b\to i} p_{k^b} \mathbb{1}\left(i \geq k^b+2\right) - e^{-\lambda\gamma\mathscr{T}} p_i(\mathscr{T}) + B_{i,N_{\mathscr{T}}}(\lambda\gamma) \tag{30}$$

which is a weighting factor related to the probability mass of that i is the starting degree, $p_i(0)$, the transition probability from the burst occurring degree k^b to i, $\frac{\widehat{k^b}}{c_{\lambda\gamma}\gamma} p_{k^b\to i}$, and the probability mass at burst occurring degree k^b, p_{k^b}; and

$$p^s_{ki} = c_{\lambda\gamma}\frac{\gamma}{\hat{k}} \prod_{j=i}^{k} \frac{\hat{j}}{\gamma+\hat{j}} \tag{31}$$

which is the degree distribution when the starting degree is i; and

$$d_{ki} = \begin{cases} p_{k^b\to k^b+1} & i \leq k^b \leq k-1 \\ 1 & k^b \leq i-1 \text{ or } k^b \geq k \end{cases} \tag{32}$$

which is a discount factor of the degree distribution when the starting degree is i, due to certain proportion of probability $\left(1-p_{k^b\to k^b+1}\right)$ is left for bursty arrival (i.e. not single arrival).

Denote the MC model with no burst structure (i.e., $p_{k^b\to k} = 0, \forall k \geq 1$ except $p_{k^b\to k^b+1} = 1$) and the same starting degree k^s for nodes (i.e., $p_k(0) = 0, \forall k \geq 0$ and $p_{k^s}(0) = 1$) as "the default MC model".

From theorem 3, it is remarked that the following useful relationships between special cases of the MC model and their general cases.

Remark 1 *For sufficiently large $\mathscr{T}$, the term $B_{i,N_{\mathscr{T}}}(s) - e^{-s\mathscr{T}} p_i(\mathscr{T})$ can be viewed as an approximation error, which is ≈ 0, for most values of i. It is because $B_{i,N_{\mathscr{T}}}(s)$ is small (and asymptotically converges to 0 as $\mathscr{T} \to \infty$), as it comes from two parts, $\lambda\widehat{(i-1)}P_{i-1,N_{\mathscr{T}}}(s)$ and $\lambda\hat{i}P_{i,N_{\mathscr{T}}}(s)$, which vanishes as $\mathscr{T} \to \infty$ (since they are Laplace transforms of the two boundary terms $\lambda\widehat{(i-1)}p_{i-1,N_{\mathscr{T}}}(t)$, and $\lambda\hat{i}p_{i,N_{\mathscr{T}}}(t)$ in (7) which are very small as compared to other terms in equation (7).). Besides that, the other part of error $e^{-s\mathscr{T}} p_i(\mathscr{T})$ is also small (and asymptotically converges to 0 as $\mathscr{T} \to \infty$).*

Remark 2 *Degree distribution p_k under MC model with (nonsingleton) starting degree(s), but no burst is a (non-degenerated) mixture of degree distributions p_{ki} of the default MC models with various starting degree(s) i, specifically,*

$$p_k = \sum_{i=0}^{k} p_i(0)\, p_{ki}^s \tag{33}$$

where $p_i(0)$ is the mixing probability.

Remark 3 *Degree distribution under the general MC model p_k is a mixture of degree distribution of the default MC models with discounting d_{ki}. The probability mass at k need discounting when the burst location is inside the interval between starting degree i and $k-1$, specifically,*

$$p_k = \sum_{i=0}^{k} c_i q_i \frac{p_{ki}^s d_{ki}}{c_i}, \qquad \forall k \geq 0 \tag{34}$$

where $c_i q_i$ is the mixing probability, with c_i being a normalization constant such that $\sum_{i=0}^{k} \frac{p_{ki}^s d_{ki}}{c_i} = 1$. The discounting factor d_{ki} of the degree distribution p_{ki} at different starting degree i could be interpreted as follows. For a fixed starting degree i, when the starting degree $i \geq k^b + 1$, no discounting of probability mass happens, i.e., the degree distribution under general MC model is the same as that under the MC model with no burst in this regime. When the starting degree $i \leq k^b$, before the burst occurs (for $k \leq k^b$), there is no discounting of the probability mass at those k, i.e. the degree distribution under general MC model is the same as that under the MC model with no burst for those k in this regime of i; yet for $k \geq k^b + 1$ in this regime of i, discounting of the probability mass at k happens, specifically, more weighting in the mixing probability in the general MC model for those with starting degree $\geq k^b + 2$, compared to that of the MC model with no burst.

S3.4 MC generalizes Poisson and Exponential network

Theorem 4 *When $L = \mathscr{L} = \mathscr{U} = U$ in the default MC model, as $\mathscr{T} \to \infty$, it reduces to one of the two classical models respectively, for*

- *Case 1 — accounting a fixed set of nodes (starting at the same time and the same degree):*
  it reduces to the Poisson *network model;*
- *Case 2 — accounting all nodes:*
  it reduces to the exponential *network model.*

Proof In this proof, the degree distribution of the network p_k is computed under the setting $L = \mathscr{L} = \mathscr{U} = U$.

In this setting, the modified degree is given by $\hat{k} = L$, for all k. Denote the starting degree as k^s, when $\mathscr{T}$ is large enough, by lemma 2, the Laplace Transform of degree distribution of a node is obtained as follows:

$\forall k \geq k^s$,

$$\begin{aligned} P_k(s) &= \left(\frac{\lambda L}{s+\lambda L}\right)^{k-k^s} \left(\frac{1}{s+\lambda L}\right) \\ &= \frac{(\lambda L)^{k-k^s}}{(k-k^s)!} \frac{(k-k^s)!}{(s+\lambda L)^{k-k^s+1}}. \end{aligned} \tag{35}$$

and $P_k(s) = 0$, $\forall k < k^s$.

- In Case 1, the specified set of nodes for statistics collection, have the same starting time a_0, and thus have been existing in the network for the same amount of residential time $\mathscr{T} - a_0$. In other words, the probability density function of the residential-time T_i of all node i are $f_{T_i}(t) = \delta(t - (\mathscr{T} - a_0))$, where $\delta(t)$ is a Dirac-delta function. Since residential time distribution is very

simple in this case, method 1 (i.e., perform inverse Laplace Transform and then take expectation over residential time) is used to find the degree distribution in this case. The inverse Laplace transform of $P_k(s)$ yields, $\forall k \geq k^s$,

$$p_k(t) = \frac{(\lambda L)^{k-k^s}}{(k-k^s)!} t^{k-k^s} e^{-\lambda L t} = \frac{(\lambda L t)^{k-k^s} e^{-\lambda L t}}{(k-k^s)!}. \tag{36}$$

and $p_k(t) = 0$, $\forall k < k^s$, which is a Poisson distribution (with starting degree k^s) with parameter λL. Then, the degree distribution of the network of the specified set of nodes is given by $p_k = p_k(\mathscr{T} - a_0) = \frac{(\lambda L T)^{k-k^s} e^{-\lambda L T}}{(k-k^s)!}$, which is a Poisson distribution (with starting degree k^s) with parameter $\lambda L T$.
A network with Poisson node degree distribution is known as the Poisson network introduced by Erdös and Rényi[20], wherein all nodes start with degree 0. Specifically, we can generate the Poisson Network in[20] by collecting statistics of initial nodes in our model with aforementioned parameters $L = \mathscr{L} = \mathscr{U} = U$ on generating the networks, with initial nodes' degree $k^s = 0$, and with $p_k(0) = 0$ for all k except $p_0(0) = 1$.

- In Case 2, all nodes are considered in the statistical collection, according to the MC model, different node i could have different starting time a_i, and hence different residential time $\mathscr{T} - a_i$. Specifically, the MC model determines the statistical properties of the dynamics of the number of nodes in the network, which would in turn determine the distribution of the residential-time T of a randomly picked node.
As an illustration on the computation of the distribution of the residential-time T, consider a special case of the MC model where $L = \mathscr{L} = \mathscr{U} = U = 1$ as in the following lemma 5:

Lemma 5 *When $L = \mathscr{L} = \mathscr{U} = U = 1$, the residential-time T of a node has an exponential distribution with rate λ.*

Proof The network dynamics satisfy

$$\frac{d}{dt} p_n(t) = \lambda (n-1) p_{n-1}(t) - \lambda n p_n(t),$$

which is obtained by summing equation (5) over k. Note that this is a Yule process, which is shown in[18] that the residential-time of a node has an exponential distribution with rate λ. ■

The detailed derivation for the general case wherein $L = \mathscr{L} = \mathscr{U} = U$, is presented in Case 1 of lemma 10 of section S4. The residential-time T is shown to be exponentially distributed with parameter λL, abbreviated as $\exp(\lambda L)$.
Thus, method 2 for calculating the degree distribution of the network is suitable in this case (i.e., by averaging the Poisson density $p_k(t)$ in equation (36) with the residential-time distribution $\exp(\lambda L)$). The result is a special case of applying theorem 3 to the default MC model with this specific set of parameter $L = \mathscr{L} = \mathscr{U} = U$ as follows:

$$p_k = c_{\lambda L} \frac{L}{L} \prod_{j=k^s}^{k} \frac{L}{L+L} \text{ where } c_{\lambda L} \approx 1 \text{ as } \mathscr{T} \text{ is large} = \frac{1}{2}\left(\frac{1}{2}\right)^{k-k^s}, \tag{37}$$

which is a geometric distribution with parameter $\frac{1}{2}$ (with the starting degree k^s), known as the node-degree distribution of an exponential network[21], since the geometric distribution is the discrete version of the exponential distribution.
A network with Exponential node degree distribution is known as the Exponential network, wherein all nodes start with degree $k^s = 1$ in most literature[22]. Specifically, we can generate the Exponential Network[22] by collecting statistics of all nodes in our model with aforementioned parameters $L = \mathscr{L} = \mathscr{U} = U$ on generating the networks, with starting nodes' degree $k^s = 1$, and with $p_k(0) = 0$ for all k except $p_{k^s}(0) = 1$.

■

From the MC model, one could also generate a more general class of exponential distribution for the degree distribution of the network, by allowing non-singleton probability mass on the starting degree k_s of nodes, i.e., different nodes could have different starting degree (mathematically, there is no single k_s such that $p_{k_s}(0) = 1$ while $p_k(0) = 0$ for other k; or in other words, $p_k(0) > 0$, for more than one k) as shown in the following corollary.

Corollary 6 *When $L = \mathscr{L} = \mathscr{U} = U$ in the general MC model with the starting degree having (non-singleton) probability mass specified by $p_i(0), \forall i \geq 0$, as $\mathscr{T} \to \infty$, it reduces to a (nono-degenerated) mixture of discounted geometric distribution with different starting degree with mixing probability q_i, i.e.,*

$$p_k = \sum_{i=0}^{k} q_i \left(\frac{1}{2}\right)\left(\frac{1}{2}\right)^{k-i} d_{ki}. \tag{38}$$

where d_{ki} is the discount factor specified in theorem 3. When the MC model with no burst structure, $q_i = p_i(0)$ and no discounting happens, i.e. $d_{ki} = 1$ for all k and i.

Proof It is a corollary of theorem 3, with p_k in equation (37) as p^s_{ki} in theorem 3 for each starting degree i being k_s in theorem 4, as discussed in Remark 2 and Remark 3. ∎

Remark 4 *As a remark to Corollary 6, equation (38) shows that this specific setting of the MC model (under the case of no burst structure, but possibly different starting degrees) gives a degree distribution of the form of a mixture of consecutively truncated geometric distributions with parameter $\frac{1}{2}$, and the mixing probability (or the i-th truncation probability) being $p_i(0)$. It looks like the discrete analog of the hyperexponential distribution. For hyperexponential distribution, as a mixture of some exponential distributions, the empirical data generated from this distribution can be well-fitted to the distribution using the well-known Prony method (widely used in the engineering literature, e.g., in power systems[23]). However, there are still some differences between equation (38) and the hyperexponential distribution. Precisely, equation (38) is a mixture of truncated geometric distributions. A simple generalization to the discrete analog of the Prony method for parameter estimation on fitting the empirical data may not be good enough, so a further extension of the method is needed to handle the effect of truncations. Since the detailed design of this possibly optimal fitting procedure still needs further investigation, it is left for future research. In this paper, only a simple heuristic procedure is proposed for fitting data from the mixture of truncated geometric distributions (see the Fitting Methodology Section in S9 for details).*

S3.5 MC generalizes the power-law model

Theorem 7 *When $L = \mathscr{L} = 1$, $U = \mathscr{U} = \infty$ in the default MC model, as $\mathscr{T} \to \infty$, it reduces to the power-law model.*

Proof In this setting, the modified degree is given by $\hat{k} = \max\{1, k\}, \forall k \geq 0$, i.e., $\hat{k} = k$, $\forall k \geq 1$ and $\hat{0} = 1$. Denote the starting degree as k^s. Recall that the degree distribution of the network is given by $p_k = \mathbb{E}_T[p_k(t)]$, where T is the random variable denoting the residential-time of a randomly picked node. The detailed derivation of the residential-time of any specific node *, when $L = \mathscr{L}$ and $U = \mathscr{U} = \infty$, is presented in Case 2 of lemma 10 of section S4, and is shown to be exponentially distributed with rate 2λ as $\mathscr{T} \to \infty$. So method 2 of finding degree distribution p_k is applicable in this case. Apply theorem 3 to the default MC model with above parameter setting, one can get

$$\begin{aligned} p_k &= \frac{2}{\max\{1,k\}} \prod_{j=k^s}^{k} \left(\frac{\max\{1,j\}}{2 + \max\{1,j\}} \right), \\ &= \frac{2\max\{1,k^s\}(k^s+1)}{\max\{1,k\}(k+1)(\max\{1,k\}+2)}, \\ &\sim c_{k^s} k^{-3} \text{ for some constant } c_{k^s} \text{ (for each given } k^s\text{), for large enough } k, \\ &\text{i.e., a Power Law with exponent -3, with the given starting degree } k^s. \end{aligned} \tag{39}$$

∎

Similar to the case of extending Exponential network to more general class of exponential distributed network, from the MC model, one could also generate a more general class of Power-Law distribution for the degree distribution of the network, by allowing non-singleton probability mass on the starting degree k_s of nodes as shown in the following corollary.

Corollary 8 *When $L = \mathscr{L} = 1$, $U = \mathscr{U} = \infty$ in the general MC model with the starting degree having (non-singleton) probability mass specified by $p_i(0), \forall i \geq 0$, as $\mathscr{T} \to \infty$, it reduces to a (non-degenerated) mixture of discounted power-law with different starting degree with mixing probability q_i, i.e.,*

$$p_k = \sum_{i=0}^{k} q_i \frac{2\max\{1,i\}(i+1)}{\max\{1,k\}(k+1)(\max\{1,k\}+2)} d_{ki}. \tag{40}$$

where d_{ki} is the discount factor specified in theorem 3. When the MC model with no burst structure, $q_i = p_i(0)$ and no discounting happens, i.e. $d_{ki} = 1$ for all k and i.

Proof It is a corollary of theorem 3, with p_k in equation (39) as p^s_{ki} in theorem 3 for each starting degree i being k_s in theorem 7, as discussed in Remark 2 and Remark 3. ∎

Remark 5 *As a remark to Corollary 8, equation (40) show that this specific setting of the MC model (under the case of no burst structure, but possibly different starting degrees) gives a degree distribution of the form of a mixture of some consecutively truncated power-law distributions having a slope parameter* -3*, with the mixing probability being* $p_i(0)$*. The resultant distribution is still a power law with a slightly greater exponent* $-3+\varepsilon$*, where* $\varepsilon > 0$ *is a very small value.*

S3.6 MC model can explain Degree Trichotomy

Besides providing a unified framework for studying general complex networks , the proposed MC model can offer an analytical closed-form expression of the degree distribution and explain the observed three phases in empirical degree distributions in realistic networks, which is addressed in this paper for the first time in the literature.

The results are summarized as follows.

Theorem 9 *The degree distribution of the default MC model (with starting degree i) in general parameter settings is given by*

$$p_{ki} \sim \begin{cases} c \cdot \text{geom}\left(\frac{\gamma}{\gamma+L}\right) & (\text{with s.d.} = i), \quad \text{if } 0 \le k \le \mathscr{L} \\ c_{2i} \cdot \text{power-law with exponent} - (\gamma+1) & (\text{with s.d.} = k^s_{2i}), \quad \text{if } \mathscr{L} < k \le \mathscr{U} \\ c_{3i} \cdot \text{geom}\left(\frac{\gamma}{\gamma+U}\right) & (\text{with s.d.} = k^s_{3i}), \quad \text{if } \mathscr{U} < k \le N_{\mathscr{T}} \end{cases} \tag{41}$$

and the degree distribution of the general MC model is a mixture of the above distributions over various i with mixing probability q_i *and burst related discount factor* d_{ki} *as follows:*

$$p_k = \sum_{i=0}^{k} q_i p_{ki} d_{ki} \tag{42}$$

where $L \le \gamma \le L+1$*. For* $U \ll N$*, one has* $\gamma \approx L$*, while for* $U \sim N$*, one has* $\gamma \approx L+1$*;*

c, c_{2i}, c_{3i} *are normalization constant making the total probability to be 1, i.e.,* $\sum_k p_k = 1$*;*

$\left(c_{2i}, k^s_{2i}\right) = \begin{cases} \left(p_{\mathscr{L}i} \cdot \frac{L}{\gamma}, \mathscr{L}+1\right) & \text{if } i \le \mathscr{L} \\ (c, i) & \text{if } i > \mathscr{L} \end{cases}$ *and* $\left(c_{3i}, k^s_{3i}\right) = \begin{cases} \left(p_{\mathscr{U}i} \cdot \frac{\mathscr{U}}{\gamma}, \mathscr{U}+1\right) & \text{if } i \le \mathscr{U} \\ (c, i) & \text{if } i > \mathscr{U} \end{cases}$ *are the (normalization constant, starting degree) pairs for degrees at phase 2 and phase 3 (It is noted that when s.d. is outside the boundary of the phase, the probability mass function is defined as* 0 *at that phase.);*

$N_{\mathscr{T}}$ *is the network size at current time* $\mathscr{T}$*.*

With no burst structure, $q_i = p_i(0)$*, i.e. the starting degree distribution, and* $d_{ki} = 1$*.*

With burst structure, q_i *is greater than* $p_i(0)$ *by a factor proportional to the probability of transfer from* k^b *to i, i.e.,* $d_{ki} = p_{k^b \to k}$ *when* k^b *is in the interval between i and* $k-1$*; and* $d_{ki} = 1$ *otherwise.*

Proof In this setting, the modified degree is given by

$$\hat{k} = \begin{cases} L, & \text{if } 0 \le k \le \mathscr{L} \\ k, & \text{if } \mathscr{L} < k \le \mathscr{U} \\ U, & \text{if } \mathscr{U} < k < N_{\mathscr{T}} \end{cases}. \tag{43}$$

Denote the starting degree as i. Recall that the degree distribution of the network is given by $p_k = \mathbb{E}_T\left[p_k(t)\right]$, where T is the random variable denoting the residential-time of a randomly picked node. As can be seen from lemma 10 of section S4, the residential-time of the node * has an exponential distribution with parameter $\lambda\gamma$ and a normalization constant $c = \frac{1}{1-e^{-\lambda\gamma\mathscr{T}}}$. So method 2 of finding degree distribution p_k is applicable in this case. Apply theorem 3 to the general MC model with above parameter setting, one can get equation (29), i.e., $p_k = \sum_{i=0}^{k} q_i p^s_{ki} d_{ki}$ $\forall k \ge 0$ with q_i given by equation (30), and p^s_{ki} and d_{ki} given as follows.

It is noted that p^s_{ki} is the degree distribution under the default MC model with starting degree i, whenever $i \le k$. And one may extend it to a matrix with row index k and column index i by setting it to be 0 for $i > k$. The specific form of the entries depends on which of the three regions that the index i and k falls in. Specifically, the matrix p^s_{ki} is a lower triangular matrix, whose entries is written in the following form:

(k,i) entries	$0 \leq i \leq \mathscr{L}$	$\mathscr{L} \leq i \leq \mathscr{U}$	$i > \mathscr{U}$
$0 \leq k \leq \mathscr{L}$	$c \cdot \frac{\gamma}{L} \left(\frac{L}{\gamma+L} \right)^{k-i+1}$	0	0
$\mathscr{L} < k \leq \mathscr{U}$	$c \cdot \frac{\gamma}{k} \frac{\frac{(k)!}{(\mathscr{L})!}}{\frac{\Gamma(\gamma+k+1)}{\Gamma(\gamma+\mathscr{L}+1)}} \left(\frac{L}{\gamma+L} \right)^{\mathscr{L}-i}$	$c \cdot \frac{\gamma}{k} \frac{\frac{(k)!}{(i-1)!}}{\frac{\Gamma(\gamma+k+1)}{\Gamma(\gamma+i)}}$	0
$k > \mathscr{U}$	$c \cdot \frac{\gamma}{U} \left(\frac{U}{\gamma+U} \right)^{k-\mathscr{U}} \frac{\frac{(\mathscr{U})!}{(\mathscr{L})!}}{\frac{\Gamma(\gamma+\mathscr{U}+1)}{\Gamma(\gamma+\mathscr{L}+1)}} \left(\frac{L}{\gamma+L} \right)^{\mathscr{L}-i}$	$c \cdot \frac{\gamma}{U} \left(\frac{U}{\gamma+U} \right)^{k-\mathscr{U}} \frac{\frac{(\mathscr{U})!}{(i-1)!}}{\frac{\Gamma(\gamma+\mathscr{U}+1)}{\Gamma(\gamma+i)}}$	$c \cdot \frac{\gamma}{U} \left(\frac{U}{\gamma+U} \right)^{k-i+1}$

where c is the normalization constant such that the sum of the probabilities is 1.

Based on the asymptotic property of the Gamma function, i.e., $\lim_{k\to\infty} \frac{\Gamma(k)k^{(\gamma+1)}}{\Gamma(k+\gamma+1)} = 1$, thus the (k,i) entries for phase 2's degrees k (i.e., $\mathscr{L} < k \leq \mathscr{U}$) would be $\gamma \frac{\Gamma(k)}{\Gamma(k+\gamma+1)} \frac{\Gamma(\gamma+(i-1)+1)}{(i-1)\Gamma(i-1)} \approx \gamma \frac{k^{-(\gamma+1)}}{(i-1)^{-(\gamma)}} \approx \frac{k^{-(\gamma+1)}}{\sum_{k=i}^{\infty} k^{-(\gamma+1)}}$, which is a power law with exponent $-(\gamma+1)$ with starting degree (s.d.) $k^s_{2i} = \begin{cases} \mathscr{L}+1 & \text{if } i \leq \mathscr{L} \\ i & \text{if } i > \mathscr{L} \end{cases}$, whenever $\mathscr{L} < k \leq \mathscr{U}$.

In summary, one can obtain p_{ki} for the degree distribution of the starting degree i as stated in the theorem as follows:

$$p_{ki} \sim \begin{cases} c \cdot \text{geom}\left(\frac{\gamma}{\gamma+L}\right) & \text{(with s.d.} = i), & \text{if } 0 \leq k \leq \mathscr{L} \\ c_{2i} \cdot \text{power-law with exponent} -(\gamma+1) & \text{(with s.d.} = k^s_{2i}), & \text{if } \mathscr{L} < k \leq \mathscr{U} \\ c_{3i} \cdot \text{geom}\left(\frac{\gamma}{\gamma+U}\right) & \text{(with s.d.} = k^s_{3i}), & \text{if } \mathscr{U} < k \leq N_{\mathscr{T}} \end{cases} \tag{44}$$

where $(c_{2i}, k^s_{2i}) = \begin{cases} \left(p_{\mathscr{L}i} \cdot \frac{L}{\gamma}, \mathscr{L}+1\right) & \text{if } i \leq \mathscr{L} \\ (c,i) & \text{if } i > \mathscr{L} \end{cases}$ and $(c_{3i}, k^s_{3i}) = \begin{cases} \left(p_{\mathscr{U}i} \cdot \frac{\mathscr{U}}{\gamma}, \mathscr{U}+1\right) & \text{if } i \leq \mathscr{U} \\ (c,i) & \text{if } i > \mathscr{U} \end{cases}$ are the (normalization constant, starting degree) pairs for degrees at phase 2 and phase 3.

For the case of the MC model with bursty structure k^b, one further need the discount matrix d_{ki}, by equation (32) in theorem 3, and apply it to the case with the above "degree-trichotomy" setting, its representation is divided into the following cases:

When $0 \leq k^b \leq \mathscr{L}$, the matrix d_{ki} is defined as

(k,i) entries	$0 \leq i \leq \mathscr{L}$	$\mathscr{L} < i \leq \mathscr{U}$	$i > \mathscr{U}$
$0 \leq k \leq \mathscr{L}$	$\begin{cases} p_{k^b \to k} & \text{if } i \leq k^b \text{ and } k^b \leq k-1 \\ 1 & \text{otherwise} \end{cases}$	1	1
$\mathscr{L} < k \leq \mathscr{U}$	$\begin{cases} p_{k^b \to k} & \text{if } k^b \leq k-1 \\ 1 & \text{otherwise} \end{cases}$	1	1
$k > \mathscr{U}$	$\begin{cases} p_{k^b \to k} & \text{if } k^b \leq k-1 \\ 1 & \text{otherwise} \end{cases}$	1	1

When $\mathscr{L} < k^b \leq \mathscr{U}$, the matrix d_{ki} is defined as

(k,i) entries	$0 \leq i \leq \mathscr{L}$	$\mathscr{L} < i \leq \mathscr{U}$	$i > \mathscr{U}$
$0 \leq k \leq \mathscr{L}$	1	1	1
$\mathscr{L} < k \leq \mathscr{U}$	$\begin{cases} p_{k^b \to k} & \text{if } i \leq k^b \text{ and } k^b \leq k-1 \\ 1 & \text{otherwise} \end{cases}$	$\begin{cases} p_{k^b \to k} & \text{if } i \leq k^b \text{ and } k^b \leq k-1 \\ 1 & \text{otherwise} \end{cases}$	1
$k > \mathscr{U}$	$p_{k^b \to k}$	$\begin{cases} p_{k^b \to k} & \text{if } i \leq k^b \\ 1 & \text{otherwise} \end{cases}$	1

When $\mathscr{U} < k^b$, the matrix d_{ki} is defined as

(k,i) entries	$0 \leq i \leq \mathscr{L}$	$\mathscr{L} < i \leq \mathscr{U}$	$i > \mathscr{U}$
$0 \leq k \leq \mathscr{L}$	1	1	1
$\mathscr{L} < k \leq \mathscr{U}$	1	1	1
$k > \mathscr{U}$	$\begin{cases} p_{k^b \to k} & \text{if } k^b \leq k-1 \\ 1 & \text{otherwise} \end{cases}$	$\begin{cases} p_{k^b \to k} & \text{if } k^b \leq k-1 \\ 1 & \text{otherwise} \end{cases}$	$\begin{cases} p_{k^b \to k} & \text{if } i \leq k^b \text{ and } k^b \leq k-1 \\ 1 & \text{otherwise} \end{cases}$

As a result, the degree distribution p_k could be view as a matrix-vector multiplication, with the matrix being the result of the matrix point-wise product of the matrix p_{ki} and one of the above d_{ki} matrices (the choice depends on k^b); and the vector being q_i given in equation (30).

Hence the result as stated in the theorem is obtained, with p_k being a mixture of discounted p_{ki} with different stating degree i, with discounting given by d_{ki} and the mixing probability given by $p_i(0)$ adjusted by the probability come from the burst.

By checking the matrix vector multiplication of $p_{ki}d_{ki}$ and q_i, and notice from Remark 3, the mixing probability governed by q_i in the general MC model have more weighting for those with starting degree $i \geq k^b + 2$ than $q_i = p_i(0)$ in the default MC model, some interpretations of the above mathematical results on the effect of burstiness are given as follows,

- Case 1: $0 \leq k^b \leq \mathscr{L}$
The resultant degree distribution has more mixing of geometric distribution in region 1 ($0 \leq k \leq \mathscr{L}$) for those $k > k^b$, and give stronger power-law (i.e., smaller power-law exponent or change from geometric to heavier-tail as in power-law) in the whole second region ($\mathscr{L} < k \leq \mathscr{U}$) and the whole third region ($k > \mathscr{U}$).
- Case 2: $\mathscr{L} < k^b \leq \mathscr{U}$
The resultant degree distribution is a stronger power-law (i.e., smaller power-law exponent or change from geometric to heavier-tail as in power-law) in the second region ($\mathscr{L} < k \leq \mathscr{U}$) for those $k > k^b$, and the third region ($k > \mathscr{U}$).
- Case 3: $k^b > \mathscr{U}$
The resultant degree distribution is more geometric in the third region ($k > \mathscr{U}$) for those $k > k^b$.

∎

It is noted in accounting the degree distribution p_k in theorem 9, there is an approximation error term in the mixing probability q_i, for very large starting degree i (corresponding to the approximation error term in $P_i(s)$ for very large i, i.e., $B_{i,N_{\mathscr{T}}}(s) - e^{-s\mathscr{T}} p_i(\mathscr{T})$), it is $B_{i,N_{\mathscr{T}}}(\lambda\gamma) - e^{-\lambda\gamma\mathscr{T}} p_i(\mathscr{T})$. It will cause some small discrepancies in observed probability density against the theoretical expression of the probability mass function under default MC model in equation (41) for very large k, even when the starting degree is a singleton k^s (as those error terms in those q_i are mainly located at very large i, which only take effect for large degree k as shown in equation (42)). This component of approximation error explains part of the errors in the so-called "node dynamics" in the existing literature for the power-law case. More details on this node-dynamics will be discussed in Section S8 on further discussion on the Simulation.

S4 A Technical lemma to derive the main theorem and its proof: Residential-time distribution of a node in the proposed MC model

In the analysis of the node-degree distribution p_k, it is very important to know how long (the residential-time T) the specified node * has been existing in the network. Denote the probability density function of the residential-time by $f_T(t)$. At the observation time $\mathscr{T}$, the node-degree distribution p_k can be written as follows:

$$p_k = \mathbb{E}_{\mathscr{T}}[p_k(t)] = \int_0^{\mathscr{T}} p_k(t) f_T(t)\, dt. \tag{45}$$

An illustration of the residential-time T of a node is shown by Fig. 1.

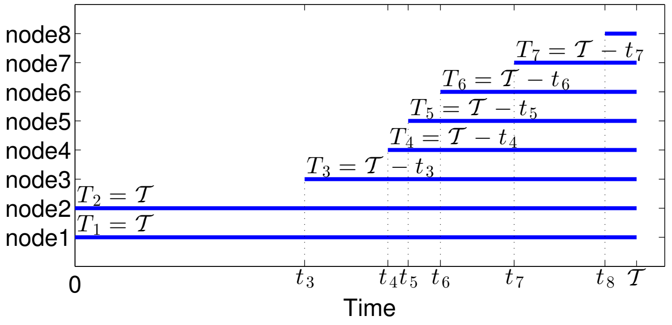

Figure 1. Arrival time of node i is denoted by t_i, and residential-time of node i is denoted by T_i, which is the same as $\mathscr{T} - t_i$, where $\mathscr{T}$ is the system starting time since the appearance of the first two nodes.

In this section, we will prove the following technical lemma on the residential-time T of a node as follow:

Lemma 10 *This residential-time probability density function $f_T(t)$ of each node in the network, under certain typical setting of the system parameters L, $\mathscr{L}$, U, and $\mathscr{U}$, is approximately given by:*

- *Case 1 — When $\mathscr{L}=1$ and $\mathscr{U}=\infty$, $f_T(t)=\frac{2\lambda e^{-2\lambda t}}{1-e^{-2\lambda\mathscr{T}}}$*
- *Case 2 — When U is large (comparable to N), $f_T(t)=\frac{\lambda(L+1)e^{-\lambda(L+1)t}}{1-e^{-\lambda(L+1)\mathscr{T}}}$.*
- *Case 3 — When $U \ll N$, $f_T(t)=\frac{\lambda Le^{-\lambda Lt}}{1-\lambda Le^{-\lambda L\mathscr{T}}}$.*

Proof This residential-time distribution $f_T(t)$ of each node in the network, in turn, is related to how the network size $N(t)$ is evolving over time t. This evolution of $N(t)$ (or specifically, the differential-difference equation on $p_n(t)$, or the marginal distribution of $N(t)$) can be obtained by summing equation (5) over k, as follows:

$$\frac{d}{dt}p_n(t)=\lambda S_{n-1}p_{n-1}(t)-\lambda S_n p_n(t). \tag{46}$$

Clearly, equation (46) is an inhomogeneous birth process.

Note that the difference between S_n and S_{n-1} is given by

$$S_n=\sum_{\substack{i=1\\ i\neq i'}}^{n-1}\hat{k}_i+\widehat{k_{i'}+1}+\hat{k}_n=S_{n-1}+\left(\widehat{k_{i'}+1}-\widehat{k_{i'}}\right)+\hat{k}_n$$

It can be verified that the difference S_n-S_{n-1} is given by $\left(\widehat{k_{i'}+1}-\widehat{k_{i'}}\right)+\hat{k}_n$. It means that when a new node comes to the network, it will create a new link and increase the rate of birth in the process by $\lambda\left(L+\varepsilon_{i'}\right)$, where $\varepsilon_{i'}$ depends on the existing degree $k_{i'}$ of node i'. Specifically,

$$\varepsilon_{i'}=\begin{cases}0 & \text{if } k_{i'}<\mathscr{L}\\ \mathscr{L}+1-L & \text{if } k_{i'}=\mathscr{L}\\ 1 & \text{if } \mathscr{L}<k_{i'}<\mathscr{U}\\ U-\mathscr{U} & \text{if } k_{i'}=\mathscr{U}\\ 0 & \text{if } k_{i'}>\mathscr{U}\end{cases}.$$

It is remarked that the following $\tilde{\varepsilon}_{i'}$ will give the same effect as the above $\varepsilon_{i'}$ (even when $\mathscr{L}\neq L$ or $\mathscr{U}\neq U$) in analyzing the dynamics on $p_n(t)$:

$$\tilde{\varepsilon}_{i'}=\begin{cases}0 & \text{if } k_{i'}<L\\ 1 & \text{if } L\leq k_{i'}<U\\ 0 & \text{if } k_{i'}\geq U\end{cases}.$$

Specifically, this sum of $\tilde{\varepsilon}_{i'}$ (i.e., $\sum_{i' \text{ is incident by a new node}}\tilde{\varepsilon}_{i'}$) gives an upper, but accurate enough, estimate of the sum $\sum_{i' \text{ is incident by a new node}}\varepsilon_{i'}$.

Since this $\varepsilon_{i'}$ depends on the current network size n, it can be viewed as a realization of a random variable ε^n, with $\varepsilon^0=0$ and $\varepsilon^1=0$, because no other node i' appears in these cases. Hence, expressing S_n and S_{n-1} by $\varepsilon_{i'}$, and replacing the sum of $\varepsilon_{i'}$ by the sum of $\tilde{\varepsilon}_{i'}$, the node dynamics (46) satisfy

$$\begin{aligned}&\frac{d}{dt}p_n(t)\\ &=\lambda\left[L(n-1)+\sum_{m=1}^{n-1}\varepsilon^{m-1}\right]p_{n-1}(t)-\lambda\left[Ln+\sum_{m=1}^{n}\varepsilon^{m-1}\right]p_n(t).\end{aligned} \tag{47}$$

Let the final network size at time $\mathscr{T}$ be $N_{\mathscr{T}}$ and consider several important cases and their network-size dynamics over time, as follows:

Case 1. When $\mathscr{L}=1$ and $\mathscr{U}=\infty$, the node dynamics satisfy

$$\begin{aligned}&\frac{d}{dt}p_n(t)\\ &=\lambda\left[(n-1)+(n-3)\right]p_{n-1}(t)-\lambda\left[n+(n-2)\right]p_n(t)\\ &=2\lambda(n-2)p_{n-1}(t)-2\lambda(n-1)p_n(t).\end{aligned}$$

In this case, the density function $f_T(t)$ of the residential-time of the specified node * is a (scaled) exponential distribution with parameter 2λ, specifically with probability density function $f_T(t) = \frac{2\lambda e^{-2\lambda t}}{1-e^{-2\lambda \mathscr{T}}}$.

Case 2. When U is large (comparable to N), the network size dynamics satisfy

$$\begin{aligned}&\frac{d}{dt}p_n(t)\\&\sim \lambda\left[L(n-1)+(n-1-c)\right]p_{n-1}(t)-\lambda\left[Ln+(n-c)\right]p_n(t)\\&=\lambda\left((L+1)(n-1)-(c)\right)p_{n-1}(t)-\lambda\left((L+1)n-(c)\right)p_n(t)\end{aligned}$$

for some $L+1 \le c \le n-1$.

According to the preferential attachment mechanism, a new node has a higher probability to connect to an existing one with a larger degree, instead of those with degrees less than or equal to L. Hence, c is small compared to n, i.e., around $L+1$, instead around $n-1$.

In this case, the density function $f_T(t)$ of the residential-time of the specified node * is given by a (scaled) exponential distribution with parameter close to $\lambda(L+1)$, specifically with probability density function $f_T(t) = \frac{\lambda(L+1)e^{-\lambda(L+1)t}}{1-e^{-\lambda(L+1)\mathscr{T}}}$.

Case 3. This is the most practical scenario. When $U \ll N$, the network size dynamics satisfy

$$\frac{d}{dt}p_n(t) \sim \lambda\left[L(n-1)+c'\right]p_{n-1}(t)-\lambda\left[Ln+c'\right]p_n(t)$$

where c' is small, compared to n, and satisfies $0 \le c' \le n-1-(L+1)$.

In this case, the density function $f_T(t)$ of the residential-time of the specified node * is a (scaled) exponential distribution with parameter close to λL, specifically with the probability density function $f_T(t) = \frac{\lambda L e^{-\lambda L t}}{1-\lambda L e^{-\lambda L \mathscr{T}}}$.

It is noted that in all above cases, the denominator of the density function $f_T(t)$ would only result in a difference in the normalization constant in the calculation of p_k in equation (45). ∎

S5 Z-Transform of Node-Degree Distribution

In this and the next section, we will derive some performance metrics , based on our result of the closed-form degree distribution.

In this section, our goal is to establish a differential equation of the generator function, or z-transform of the node-degree distribution p_k. The z-transform of the degree distribution is widely used in deriving many network metrics[24], e.g., the average path length, and some closeness measures, such as clustering coefficient.

Define the generating function (also called the z-transform) of the degree distribution p_{ki} (with starting degree i) as

$$\mathbb{P}_i(z) \triangleq \sum_{k=0}^{N_{\mathscr{T}}} p_{ki} z^k. \tag{48}$$

By equation (44), it turns out $\mathbb{P}(z)$, depending on the starting degree i, can be split into three possible terms as follows:

$$\mathbb{P}_i(z) = \begin{cases}\mathbb{P}_{(i,\mathscr{L})}(z)+\mathbb{P}_{(\mathscr{L}+1,\mathscr{U})}(z)+\mathbb{P}_{(\mathscr{U}+1,N_{\mathscr{T}})}(z) & \text{if } 0 \le i \le \mathscr{L}\\ \mathbb{P}_{(i,\mathscr{U})}(z)+\mathbb{P}_{(\mathscr{U}+1,N_{\mathscr{T}})}(z) & \text{if } \mathscr{L} < i \le \mathscr{U}\\ \mathbb{P}_{(i,N_{\mathscr{T}})}(z) & \text{if } \mathscr{U} < i \le N_{\mathscr{T}}\end{cases} \tag{49}$$

where

$$\mathbb{P}_{(k',\mathscr{L})}(z) \triangleq \sum_{k=k'}^{\mathscr{L}} p_{ki} z^k = c \cdot \frac{\gamma}{L}\left(\frac{L}{\gamma+L}\right)^{\max\left\{k'-i,0\right\}+1} z^{\max\left\{k',i\right\}} \frac{1-\left(\frac{Lz}{\gamma+L}\right)^{\mathscr{L}-\max\left\{k',i\right\}+1}}{1-\frac{Lz}{\gamma+L}} \mathbb{1}\left(i \le \mathscr{L}\right), \qquad \text{for } 0 \le k' \le \mathscr{L} \tag{50}$$

$$\mathbb{P}_{(k',\mathscr{U})}(z) \triangleq \sum_{k=k'}^{\mathscr{U}} p_{ki} z^k = c_{2i} \cdot \sum_{k=\max\{k',i\}}^{\mathscr{U}} \frac{\gamma}{k} \prod_{j=k_{2i}^s}^{k} \frac{j}{\gamma+j} z^k \mathbb{1}\left(i \le \mathscr{U}\right), \qquad \text{for } \mathscr{L} < k' \le \mathscr{U} \tag{51}$$

$$\mathbb{P}_{(k',N_{\mathscr{T}})}(z) \triangleq \sum_{k=k'}^{N_{\mathscr{T}}} p_{ki} z^k = c_{3i} \cdot \frac{\gamma}{U}\left(\frac{U}{\gamma+U}\right)^{\max\left\{k'-k_{3i}^s,0\right\}+1} z^{\max\left\{k',i\right\}} \frac{1-\left(\frac{Uz}{\gamma+U}\right)^{N_{\mathscr{T}}-\max\left\{k',i\right\}+1}}{1-\frac{Uz}{\gamma+U}}, \qquad \text{for } \mathscr{U} < k' \le N_{\mathscr{T}} \tag{52}$$

S6 Complementary Cumulative Distribution Function of Node-Degree

We will use the result in last section S5 to derive the Complementary Cumulative Distribution Function (ccdf) of Node-Degree for the case of starting degree i, i.e. $F^c_{ki} \triangleq \sum_{j=k+1}^{N_{\mathscr{T}}} p^s_{ji}$. And according to equations (48), the ccdf could be rewritten as

$$F^c_{ki} = \begin{cases} \mathbb{P}_{(k+1,\mathscr{L})}(1) + \mathbb{P}_{(\mathscr{L}+1,\mathscr{U})}(1) + \mathbb{P}_{(\mathscr{U}+1,N_{\mathscr{T}})}(1) & \text{if } 0 \le k+1 \le \mathscr{L} \\ \mathbb{P}_{(k+1,\mathscr{U})}(1) + \mathbb{P}_{(\mathscr{U}+1,N_{\mathscr{T}})}(1) & \text{if } \mathscr{L} < k+1 \le \mathscr{U} \\ \mathbb{P}_{(k+1,N_{\mathscr{T}})}(1) & \text{if } \mathscr{U} < k+1 \le N_{\mathscr{T}} \end{cases} \tag{53}$$

where

$$\mathbb{P}_{(k+1,\mathscr{L})}(1) = c \cdot \frac{\gamma}{L}\left(\frac{L}{\gamma+L}\right)^{\max\{k+1-i,0\}+1} \frac{1-\left(\frac{L}{\gamma+L}\right)^{\mathscr{L}-\max\{k+1,i\}+1}}{1-\frac{L}{\gamma+L}} \mathbb{1}\left(i \le \mathscr{L}\right), \qquad \text{for } 0 \le k+1 \le \mathscr{L} \tag{54}$$

$$\mathbb{P}_{(k+1,\mathscr{U})}(1) = c_{2i} \cdot \sum_{l=\max\{k+1,i\}}^{\mathscr{U}} \frac{\gamma}{l} \prod_{j=k^s_{2i}}^{l} \frac{j}{\gamma+j} \mathbb{1}\left(i \le \mathscr{U}\right), \qquad \text{for } \mathscr{L} < k+1 \le \mathscr{U} \tag{55}$$

$$\mathbb{P}_{(k+1,N_{\mathscr{T}})}(1) = c_{3i} \cdot \frac{\gamma}{U}\left(\frac{U}{\gamma+U}\right)^{\max\left\{k+1-k^s_{3i},0\right\}+1} \frac{1-\left(\frac{U}{\gamma+U}\right)^{N_{\mathscr{T}}-\max\{k+1,i\}+1}}{1-\frac{U}{\gamma+U}}, \qquad \text{for } \mathscr{U} < k+1 \le N_{\mathscr{T}} \tag{56}$$

With some simplification, we noted that

$$\mathbb{P}_{(\mathscr{U}+1,N_{\mathscr{T}})}(1) = \begin{cases} p_{\mathscr{U}i} \cdot \frac{\mathscr{U}}{\gamma} \cdot \frac{\gamma}{U}\left(\frac{U}{\gamma+U}\right) \frac{1-\left(\frac{U}{\gamma+U}\right)^{N_{\mathscr{T}}-\mathscr{U}}}{1-\frac{U}{\gamma+U}}, & \text{for } 0 \le i \le \mathscr{U} \\ c \cdot \frac{\gamma}{U}\left(\frac{U}{\gamma+U}\right) \frac{1-\left(\frac{U}{\gamma+U}\right)^{N_{\mathscr{T}}-i+1}}{1-\frac{U}{\gamma+U}}, & \text{for } \mathscr{U} < i \le N_{\mathscr{T}} \end{cases} \tag{57}$$

and

$$\mathbb{P}_{(\mathscr{L}+1,\mathscr{U})}(1) = \begin{cases} p_{\mathscr{L}i} \cdot \frac{L}{\gamma} \cdot \sum_{l=\mathscr{L}+1}^{\mathscr{U}} \frac{\gamma}{l} \prod_{j=\mathscr{L}+1}^{l} \frac{j}{\gamma+j}, & \text{for } 0 \le i \le \mathscr{L} \\ c \cdot \sum_{l=i}^{\mathscr{U}} \frac{\gamma}{l} \prod_{j=i}^{l} \frac{j}{\gamma+j}, & \text{for } \mathscr{L} < i \le \mathscr{U} \\ 0 & \text{for } \mathscr{U} < i \le N_{\mathscr{T}} \end{cases} \tag{58}$$

Thus, for $0 \le i \le \mathscr{L}$

$$F^c_{ki} = \begin{cases} \mathbb{P}_{(k+1,\mathscr{L})}(1) + p_{\mathscr{L}i} \cdot \frac{L}{\gamma} \cdot \sum_{l=\mathscr{L}+1}^{\mathscr{U}} \frac{\gamma}{l} \prod_{j=\mathscr{L}+1}^{l} \frac{j}{\gamma+j} + p_{\mathscr{U}i} \cdot \frac{\mathscr{U}}{\gamma} \cdot \frac{\gamma}{U}\left(\frac{U}{\gamma+U}\right) \frac{1-\left(\frac{U}{\gamma+U}\right)^{N_{\mathscr{T}}-\mathscr{U}}}{1-\frac{U}{\gamma+U}} & \text{if } 0 \le k+1 \le \mathscr{L} \\ p_{\mathscr{L}i} \cdot \frac{L}{\gamma} \cdot \sum_{l=k+1}^{\mathscr{U}} \frac{\gamma}{l} \prod_{j=\mathscr{L}+1}^{l} \frac{j}{\gamma+j} + p_{\mathscr{U}i} \cdot \frac{\mathscr{U}}{\gamma} \cdot \frac{\gamma}{U}\left(\frac{U}{\gamma+U}\right) \frac{1-\left(\frac{U}{\gamma+U}\right)^{N_{\mathscr{T}}-\mathscr{U}}}{1-\frac{U}{\gamma+U}} & \text{if } \mathscr{L} < k+1 \le \mathscr{U} \\ p_{\mathscr{U}i} \cdot \frac{\mathscr{U}}{\gamma} \cdot \frac{\gamma}{U}\left(\frac{U}{\gamma+U}\right)^{k-\mathscr{U}+1} \frac{1-\left(\frac{U}{\gamma+U}\right)^{N_{\mathscr{T}}-k}}{1-\frac{U}{\gamma+U}} & \text{if } \mathscr{U} < k+1 \le N_{\mathscr{T}} \end{cases} \tag{59}$$

where $\mathbb{P}_{(k+1,\mathscr{L})}(1)$ is given in equation (54), and for $\mathscr{L} < i \le \mathscr{U}$

$$F^c_{ki} = \begin{cases} 1 = c \cdot \sum_{l=i}^{\mathscr{U}} \frac{\gamma}{l} \prod_{j=i}^{l} \frac{j}{\gamma+j} + p_{\mathscr{U}i} \cdot \frac{\mathscr{U}}{\gamma} \cdot \frac{\gamma}{U}\left(\frac{U}{\gamma+U}\right) \frac{1-\left(\frac{U}{\gamma+U}\right)^{N_{\mathscr{T}}-\mathscr{U}}}{1-\frac{U}{\gamma+U}} & \text{if } 0 \le k+1 \le \mathscr{L} \\ c \cdot \sum_{l=\max\{k+1,i\}}^{\mathscr{U}} \frac{\gamma}{l} \prod_{j=i}^{l} \frac{j}{\gamma+j} + p_{\mathscr{U}i} \cdot \frac{\mathscr{U}}{\gamma} \cdot \frac{\gamma}{U}\left(\frac{U}{\gamma+U}\right) \frac{1-\left(\frac{U}{\gamma+U}\right)^{N_{\mathscr{T}}-\mathscr{U}}}{1-\frac{U}{\gamma+U}} & \text{if } \mathscr{L} < k+1 \le \mathscr{U} \\ p_{\mathscr{U}i} \cdot \frac{\mathscr{U}}{\gamma} \cdot \frac{\gamma}{U}\left(\frac{U}{\gamma+U}\right)^{k-\mathscr{U}+1} \frac{1-\left(\frac{U}{\gamma+U}\right)^{N_{\mathscr{T}}-k}}{1-\frac{U}{\gamma+U}} & \text{if } \mathscr{U} < k+1 \le N_{\mathscr{T}} \end{cases} \tag{60}$$

and for $\mathscr{U} < i \le N_{\mathscr{T}}$

$$F^c_{ki} = \begin{cases} 1 = c \cdot \frac{\gamma}{U}\left(\frac{U}{\gamma+U}\right)\frac{1-\left(\frac{U}{\gamma+U}\right)^{N_{\mathscr{T}}-i+1}}{1-\frac{U}{\gamma+U}} & \text{if } 0 \le k+1 \le \mathscr{L} \\ 1 = c \cdot \frac{\gamma}{U}\left(\frac{U}{\gamma+U}\right)\frac{1-\left(\frac{U}{\gamma+U}\right)^{N_{\mathscr{T}}-i+1}}{1-\frac{U}{\gamma+U}} & \text{if } \mathscr{L} < k+1 \le \mathscr{U} \\ c \cdot \frac{\gamma}{U}\left(\frac{U}{\gamma+U}\right)^{\max\{k+1-i,0\}+1}\frac{1-\left(\frac{U}{\gamma+U}\right)^{N_{\mathscr{T}}-\max\{k+1,i\}+1}}{1-\frac{U}{\gamma+U}} & \text{if } \mathscr{U} < k+1 \le N_{\mathscr{T}} \end{cases} \tag{61}$$

In summary, the following theorem is obtained:

Theorem 11 *The ccdf of the network degree under the default MC model, with starting degree i, is given by*

$$F^c_{ki} \sim \begin{cases} c \cdot F^c_{\text{geom}\left(\frac{\gamma}{\gamma+L}\right)} + F^c_{\mathscr{L},i} & (\textit{with s.d.} = i), \quad \textit{if } 0 \le k+1 \le \mathscr{L} \\ c_{2i} \cdot (\text{power-law } \textit{with exponent} - \gamma) + F^c_{\mathscr{U},i} & (\textit{with s.d.} = k^s_{2i}), \quad \textit{if } \mathscr{L} < k+1 \le \mathscr{U} \\ c_{3i} \cdot F^c_{\text{geom}\left(\frac{\gamma}{\gamma+U}\right)} & (\textit{with s.d.} = k^s_{3i}), \quad \textit{if } \mathscr{U} < k+1 \le N_{\mathscr{T}} \end{cases} \tag{62}$$

where $F^c_{\text{geom}\left(\frac{\gamma}{\gamma+L}\right)}$ and $F^c_{\text{geom}\left(\frac{\gamma}{\gamma+U}\right)}$ are ccdf of geometric distribution with parameters $\frac{\gamma}{\gamma+L}$ and $\frac{\gamma}{\gamma+U}$ respectively, and parameters definitions are same as those in theorem 9. (It is noted that when s.d. is outside the boundary of the phase, the ccdf is defined as 1 at that phase, and when $k <$ s.d., ccdf value at that k would be 1.) Similarly, the network degree distribution, i.e., cummulative distribution function (cdf), with starting degree i, is given by $F_{ki} = 1 - F^c_{ki}$.

The cummulative distribution function (cdf) of the general MC model is a mixture of above cdf F_{ki} over various i with mixing probability q_i and burst related discount factor $p_{k^b \to k^b+1}$ as follows:

$$F_k = \sum_{i=0}^{k} q_i \tilde{F}_{ki} \tag{63}$$

where $\tilde{F}_{ki} = \begin{cases} F_{k^b i} + \sum_{j=k^b}^{k} p^s_{ji} p_{k^b \to k^b+1} & \textit{if } i \le k^b \\ F_{ki} & \textit{if } i > k^b \end{cases}$. *Similarly the ccdf is given by $1 - F_k$.*

Proof

$$\begin{aligned} F_k &= \sum_{j=0}^{k} p_j = \sum_{j=0}^{k}\sum_{i=0}^{j} q_i p^s_{ji} d_{ji} = \sum_{i=0}^{k}\sum_{j=i}^{k} q_i p^s_{ji} d_{ji} \\ &= \sum_{i=0}^{k} q_i \left(F_{k^b i} + \sum_{j=k^b+1}^{k} p^s_{ij} d_{ji} \right) \\ &= \sum_{i=0}^{k} q_i \left(F_{k^b i} + \sum_{j=k^b+1}^{k} p^s_{ji} p_{k^b \to k^b+1} \right) + \sum_{i=0}^{k} q_i \left(F_{k^b i} + \sum_{j=k^b+1}^{k} p^s_{ji} \right) \\ &= \sum_{i=0}^{k} q_i \tilde{F}_{ki} \end{aligned} \tag{64}$$

∎

S7 Diameter of the Network

In this section, we want to compare the diameter of the network (i.e., the maximum distance between a pair of nodes in the network) exhibiting trichotomy in degree distribution under our Markov model and that of the scale-free network (e.g. the BA model):

We start with the first node of the network, and called it the root. For simplest illustration of the impact of setup parameter L and saturation parameter U, assume in our model $L = \mathscr{L}$, $U = \mathscr{U}$, and $p_1(0) = 1, p_k(0) = 0$ for all other k, i.e. only one

edge is formed when a new node joins the network. In this case, the resultant network would be a tree (both under our Markov model and the BA model).

Traversing along each edge attached to the root, some nodes are farthest from the root (i.e., within each branch of the tree, there is a node takes the maximum number of hops from the root to it). Collect all those nodes from all branch of the root and call them the foremost nodes. Then the problem of computing the diameter of the network can be reduced to the maximum distance between a pair of foremost nodes (it is remarked that this pair of foremost nodes may not be unique). We call the foremost nodes achieving this maximum distance as the candidate foremost nodes. It is noted that only attachment of a new node to one of the candidate foremost nodes will increase the diameter of the network. Hence, when a new node arrives at the time when the network size is n, the probability of increasing the diameter would be the same as the probability of attaching it to one of the candidate foremost node. Summing these diamater increasing events' probabilities from $n = 1$ to the final system size $N_{\mathscr{T}}$ would give us the expected network diameter at observation time $\mathscr{T}$.

According to the attachment rule of our Markov model, the probability of attaching the new node to one of the candidate foremost node, would be proportional to the modified degree of the candidate foremost node. Specifically, this probability would be given by the ratio of the modified degree of the candidate foremost node to the total modified degrees, times the number of candidate foremost nodes

Since each candidate foremost node has degree one,

- for our MC model, its modified degree is L,
- for BA model, its modified degree is still 1.

When the network size is n, the sum of modified degrees of all nodes in the network are also different for each model,

- for our MC model, it is $N_{L,n}L + \sum_{\{i|k_i=\hat{k}_i\}} k_{i,n} + N_{U,n}U$, where $N_{L,n}$ = the number of nodes in the set-up phase, and $N_{U,n}$ = the number of nodes in the saturation phase. It is noted that the number of nodes within power-law regime is given by $\left|\left\{i \mid k_i = \hat{k}_i\right\}\right| = n - N_{L,n} - N_{U,n}$.
- for BA model, it is the total degree and is given by $2n$.

When the network size is n, denote the number of candidate foremost nodes c_n as:

- $c_{Markov,n}$ for our Markov model, in general case; and
- $c_{BA,n}$ for B-A model, i.e. $L = 1, U = \infty$ under our model.

It is noted that as the network size n increases, c_n in both cases would stay the same whenever a new node is attached to an internal node (not connecting to candidate foremost nodes) of the tree, which happens most of the time when network size is large. Occasionally, the list of candidate foremost nodes can also keep expanding or potentially increase in next step (when a new node is attached to a leaf node of the tree which is not the candidate foremost node or attached to a sibling node of the candidate foremost node), and c_n can also decrease whenever a new node is attached to one of the candidate foremost nodes. Since when the network size is large, most of the nodes are internal nodes, those new node attachment events causing the changes in c_n are relatively rare compared to those events causing no changes in c_n. Hence c_n stay the same most of the time, even though it can fluctuate between 2 and a small maximum value, as n increases (as expected and observed in simulation). For simplicity of analysis, let us treat c_n as a $\Theta(1)$ constant, and treat it as roughly the same for both cases.

As a result, the probability of increasing the diameter at the time when the network size is n, is given by the following:

- for our Markov model, it is
$$\frac{c_{Markov,n}L}{2n + \sum_{i=1}^{N_{L,n}} (L - k_i)\mathbb{1}(k_i \leq L) - \sum_{i=1}^{N_{U,n}} (k_i - U)\mathbb{1}(k_i \geq U)};$$
- for B-A model, it is $\frac{c_{BA,n}}{2n}$.

Hence, an estimate on the diameter when network size is $N_{\mathscr{T}}$ is given by:

- Under our Markov Model, the value is:

$$\sum_{n=1}^{N_{\mathscr{T}}} \frac{c_{Markov,n}L}{N_{L,n}L + \sum_{i=1}^{n-N_{L,n}-N_{U,n}} k_{i,n} + N_{U,n}U}$$

$$= \sum_{n=1}^{N_{\mathscr{T}}} \frac{c_{Markov,n}L}{2n + \sum_{i=1}^{N_{L,n}} (L-k_i)\mathbb{1}(k_i \leq L) - \sum_{i=1}^{N_{U,n}} (k_i - U)\mathbb{1}(k_i \geq U)}; \tag{65}$$

- Under the B-A Model, the value is:

$$\sum_{n=1}^{N_{\mathscr{T}}} \frac{c_{BA,n}}{2n}. \tag{66}$$

In both cases, the estimates of the network diameters are harmonic series, so according to the property of harmonic series, the diameter scale as $\Theta(logN_{\mathscr{T}})$. However, one could have some simple observation on how L and U affect the scaling constant of this asymptotic of the network diameter.

When comparing the diameters of two models (of different L and U, a special case of $L=1$ and $U=\infty$ is the BA model), whether the "new" model has a longer diameter compared to the "old" one depends on whether (I) or (II) is larger, where

$$(I) = \frac{2n + \sum_{i=1}^{N_{L_{new},n}} (L_{new}-k_i)\mathbb{1}(k_i \leq L_{new}) - \sum_{i=1}^{N_{U_{new},n}} (k_i - U_{new})\mathbb{1}(k_i \geq U_{new})}{2n + \sum_{i=1}^{N_{L_{old},n}} (L_{old}-k_i)\mathbb{1}(k_i \leq L_{old}) - \sum_{i=1}^{N_{U_{old},n}} (k_i - U_{old})\mathbb{1}(k_i \geq U_{old})}$$

$$(II) = \left(\frac{c_{new}L_{new}}{c_{old}L_{old}}\right). \tag{67}$$

In particular, when $(I) \leq (II)$, the diameter of the new model is larger; otherwise it is smaller. As $c_{new} \approx c_{old}$, one could observe that: when U increases (for fixed L), $(I) \leq (II)$, so the diameter increases; when L increases (for fixed U), it is still more likely that $(I) \leq (II)$, so the diameter also increases.

In short, having a longer setup phase $L > 1$ and an earlier saturation phase ($U < \infty$) generally increase the diameter of the network. This analysis show that trichotomy network may not be as good as scale-free network for information dissemination if it takes a cost to traverse each hop in the network. Hence network designer aiming for faster information dissemination may consider remedies to reduce L and increase U.

S8 Further Discussion on the Simulation

S8.1 More illustrations of the degree distribution resultant from the proposed MC model

In the main text, by setting $L = \mathscr{L} = 2$ and $U = \mathscr{U} = 8$, the MC model generates the trichotomy distribution as shown in Fig.2(a), i.e., power-law distribution with exponential head and tail. Several characteristics of the node-degree distribution plot matches with those predicted by theory. As a second illustration, the same predictions also hold when $L = \mathscr{L} = 3$ and $U = \mathscr{U} = 10$, as shown in Fig. 2(b). Referring to the middle range of the closed-form expression (41), and $\gamma \approx L$ (for $\mathscr{U} \ll N$), the theory predicts that the slope is $-(L+1)$ and it matches with the observed exponent -4 in Fig. 2(b). It is also observed that the head part is geometrically distributed with parameter value 0.53 and the tail part is geometrically distributed with parameter value 0.25, as predicted by the theory.

S8.2 Discussions on the accuracy of approximation

In the main text, it is remarked that simulation curve is an averaging of the empirical distribution results over $N = 100$ simulation runs, where the goal is to reduce the variation in the empirical distribution plot, particularly reduce the variation in the tail part, so that the main characteristic of the result can be observed more easily. The reason that N can reduce the variance is because of the following asymptotic convergence theorem: sample averaged empirical degree distribution converges to the true

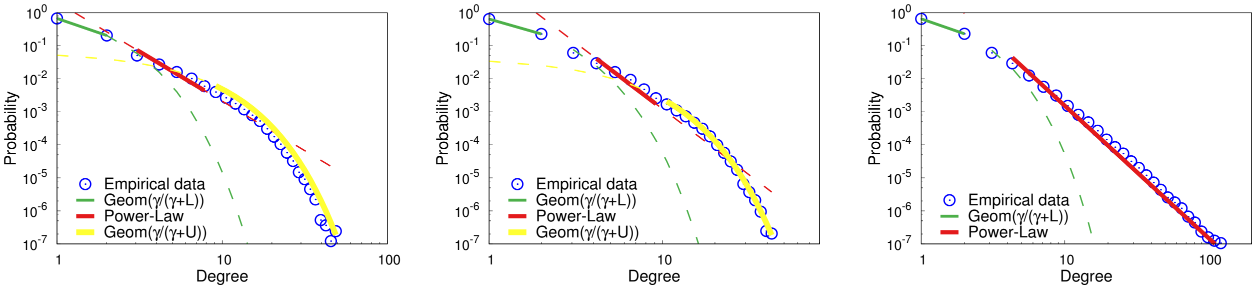

(a) $L=2, U=8$ (b) $L=3, U=10$ (c) $L=3, U=N$

Figure 2. Simulation results of node-degree distributions on the MC model under varying L and U.

degree distribution function as $N \to \infty$. Specifically, according to the strong law of large numbers and the fact that the network degree distribution $p_k = \frac{1}{N_{\mathcal{T}}} \sum_{i=1}^{N_{\mathcal{T}}} \int_0^{\mathcal{T}} p_{k_i}(t) f_{T_i}(t)\, dt$ (as mentioned before in section S3.3), the sample averaged (over simulation) empirical distribution would be asymptotically given as follows:

$$\lim_{N\to\infty} \frac{1}{N} \sum_{j=1}^{N} \left(\frac{1}{N_{\mathcal{T}}} \sum_{i=1}^{N_{\mathcal{T}}} \mathbb{1}\left\{k_i^j = k\right\} \right) = \lim_{N\to\infty} \frac{1}{N_{\mathcal{T}}} \sum_{i=1}^{N_{\mathcal{T}}} \left(\frac{1}{N} \sum_{j=1}^{N} \mathbb{1}\left\{k_i^j = k\right\} \right) \overset{a.s.}{\to} \frac{1}{N_{\mathcal{T}}} \sum_{i=1}^{N_{\mathcal{T}}} \mathbb{E}\left(\mathbb{1}\left\{k_i^j = k\right\}\right) = \frac{1}{N_{\mathcal{T}}} \sum_{i=1}^{N_{\mathcal{T}}} \mathbb{E}_{T_i} p_{k_i}(T_i) = p_k, \tag{68}$$

(where k_i^j is the degree of the i-th node at the j-th simulation at the observation time $\mathcal{T}_j$) for each k, as $N \to \infty$ (where $\mathbb{1}\{\cdot\}$ is an indicator function), i.e., the empirical distribution will converge almost surely (a.s.) to the true degree distribution. The setting on N ($N = 100$) is already large enough to ensure a small variation for most parts of the curve (i.e., for most values of k). However, there is still a significant variation in the tail part (when k is very large) of the empirical distribution observed from the simulation, especially when U is large. For example, $U = \infty$ yields the BA model, but from the proposed MC model, there is significant variation in the tail part as shown in Fig. 2(c) even if the simulation runs 100 times. These tail variations in BA model are usually called the "node dynamics" in the existing literature, which have not been theoretically addressed. In the proposed MC model, the difference between the deterministic part of the "node dynamics" and the proposed closed-form distribution, is the neglected boundary terms $\lambda\widehat{(k-1)}p_{k-1,N_T}(t)$ and $\lambda\hat{k}p_{k,N_T}(t)$ in equation (7), to obtain the degree dynamics in equation (8), which causes an approximation error $B_{k,N_{\mathcal{T}}}(\lambda\gamma) - e^{-\lambda\gamma\mathcal{T}} p_k(\mathcal{T})$ as shown in the proof of Theorem 9. It is noted that this error term is very small for small k, hence error almost only occur at the tail (i.e., large k). In particular, the larger this term $B_{k,N_{\mathcal{T}}}(\lambda\gamma)$ is, the more discrepancy it causes to the approximation formula and the empirical curves at the tail. As a result, from the proposed MC model viewpoint, there will be larger error at the tail for larger U, as $\hat{k}$ can increase further with a looser upper bound causing more discrepancy (or dynamics), while the approximation error for the model with a smaller U is smaller, as $\hat{k}$ cannot increase too far due to the tighter upper bound causing less discrepancy (or dynamics). This prediction is verified in the simulation as shown in Fig. 2(b) (where $L=3, U=10$) and Fig. 2(c) (where $L=3, U=N$).

S9 Fitting Methodology

As the degree distribution is typically provided as a list of positive integers x_{min}, ..., x_{max}, we aim to fit the data with a discrete set of data points. Let $N_{\mathcal{T}}$ denote the number of data points. The steps of the fitting process are:

Step 1. Partial logarithmic binning : Because there are much more data points in the tail than that in the head of the degree distributions which may lead the fittings to over-focus on the tails, we first compute the probability density function (PDF) using bins of exponentially increasing width $B^i | i = 0,1,2,\ldots$ when the degree x is larger than a threshold[25].

Step 2. Find $\mathscr{L}$: Choose a value of X_{min} between x_{min} and x_{max} and estimate the value of the degree exponent corresponding to this X_{min} using

$$\gamma = 1 + N_{\mathcal{T}} \left[\sum_{i=1}^{N_{\mathcal{T}}} ln \frac{x_i}{X_{min} - \frac{1}{2}} \right]^{-1}. \tag{69}$$

With the obtained parameter pair (γ, X_{min}), assume that the degree distribution has the form

$$p_x = \frac{1}{\zeta(\gamma, X_{min})} x^{-\gamma}, \tag{70}$$

where $\zeta(\gamma, X_{min}) = \sum_{n=0}^{\infty} \frac{1}{(X_{min}+n)^{\gamma}}$ is Hurwitz zeta function. Then, with the current X_{min}, use the Kormogorov-Smirnov test to determine the maximum distance $D(X_{min})$ between the CDF of the data $S(x)$ and the CDF of the estimated degree distribution $S(p_x)$:

$$D(X_{min}) = \max_{x>X_{min}} |S(x) - S(p_x)|. \tag{71}$$

We scan the whole range of X_{min} from x_{min} to x_{max} and calculate the corresponding D. We aim to identify the X_{min} value for which D is minimal

$$\mathscr{L} = \underset{X_{min}}{argmin} D(X_{min}). \tag{72}$$

Step 3. Find $\mathscr{U}$: Similar to Step 2, scan the whole X_{max} range from X_{min} to x_{max} and calculate the corresponding D:

$$D(X_{max}) = \max_{X_{min}<x\leq x_{max}} |S(x) - S(p_x)|. \tag{73}$$

We aim to identify the X_{max} value for which D is minimal:

$$\mathscr{U} = \underset{X_{max}}{argmin} D(X_{max}). \tag{74}$$

Step 4. Fit the power-law segment : We apply least-squares fitting to fit the segment $[\mathscr{L}, \mathscr{U}]$ with a power-law $p_{phase2} = a \times k^{-\gamma}$, where a is a coefficient and $-\gamma$ is the exponent.

Step 5. Fit the small-degree flattening segment (i.e., the phase exhibiting $geom(\frac{\gamma}{L+\gamma})$ distribution): With the exponent found in Step 4, we use the derived closed-form formula to fit the initializing phase. Since it is usually unclear how many truncated geometric distributions are combined in this phase, we use only two truncated geometric distributions (i.e., the maximum number of head parameter is set to 1). According to the equation, $p_{phase1} = p_1^0 \times p_a \times (1-p_a)^{k-1} \mathbb{1}_{\{k\geq 1\}} + \left(1-p_1^0\right) \times p_a \times (1-p_a)^{k-2} \mathbb{1}_{\{k\geq 2\}}$, where $p_a = \gamma/(L+\gamma)$. We then apply least-squares fitting to find the best p_1^0 so as to fit the initializing phase segment $[1, \mathscr{L}]$. For the general case of fitting the initializing phase (with a larger maximum number of head parameters), it is referred to Fig. 3.

Step 6. Fit the high-degree cutoff segment (i.e., the phase exhibiting the $geom(\frac{\gamma}{U+\gamma})$ distribution): We fit the high-degree cutoff segment by the derived closed-form formula $p_{phase3} = p_b \times (1-p_b)^{k-U-1}$, where $p_b = \gamma/(U+\gamma)$. Once γ and U are found in the previous steps, p_{phase3} is determined for the maturing phase segment $[\mathscr{U}, N_{\mathscr{T}}]$.

Step 7. Normalization : Finally, we normalize the probability of three segments to make the sum to 1. In other words, find c such that $c\left[\sum_{k=1}^{L} p_{phase1}(k) + \sum_{k=L}^{U} p_{phase2}(k) + \sum_{k=U}^{N_{\mathscr{T}}} p_{phase3}(k)\right] = 1$.

Table 3. Social Networks Datasets.

Dataset	Date	# of Nodes
DBLP Coauthor[26]	1995-2014	1,234,706 authors
Facebook Friendship[27,28]	2012	4,039 users
Twitter Friendship[27,29]	2012	81,306 users

S10 Further Discussion on the Experimental Data

The main text provides three datasets from citation networks. In this section of the Supplementary Information, another six datasets from social networks and vehicular networks are provided to verify our model.

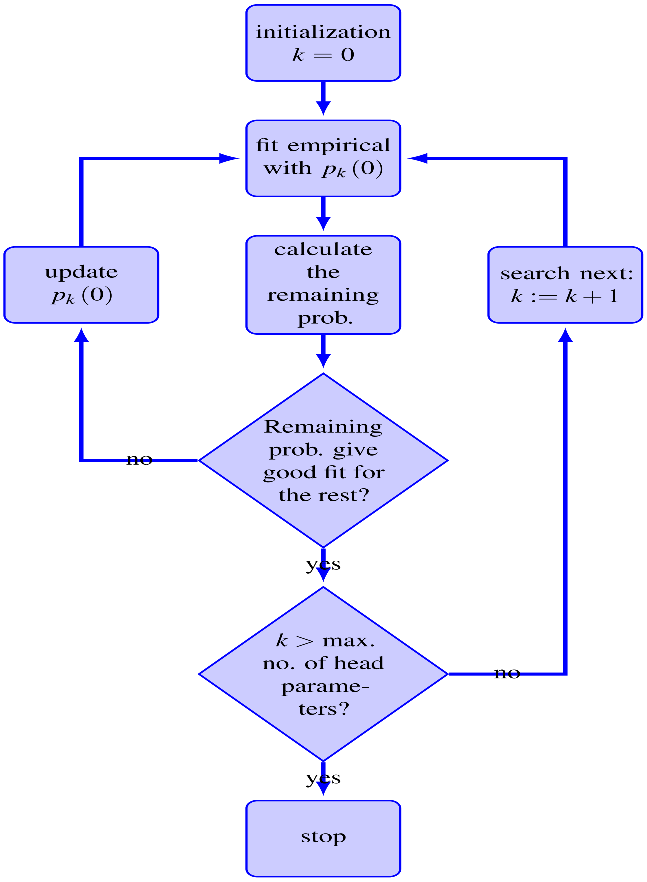

Figure 3. The method of fitting the head part of the distribution, i.e. the initializing phase

S10.1 Social networks

Table 3 shows three social network datasets[26,28,29], which are used in our verification and analysis.

MC model of Social networks: In social networks, people are considered as nodes. When a person $p1$ coauthors a paper with another person $p2$, adds $p2$ as a friend on Facebook, or follows $p2$ on Twitter, a link is built between $p1$ and $p2$ and the node degrees of both persons increase by 1. To further explain the lower bound L and the upper bound U in the new MC model, take the coauthorship network as an example. When there is no *famous scientist* (i.e., a node with high degree), each scientist likely chooses to work with others with an equal probability. When there are more and more scientists in the network, a new scientist has a higher probability to choose to work with those having better reputation (i.e., nodes with degrees $> L$). For those who are already very famous (i.e., nodes with degrees $> U$), a new scientist may choose to work with any of them with an equal probability because they all have a good enough reputation. It is a similar process for friendships in Facebook and Twitter.

Empirical Data and Fitting Results:

Fig. 4 shows the distributions of coauthors for DBLP Computer Science publications, friends in Facebook datasets, and followers in Twitter datasets. Fig. 4(b) further shows the coauthor distribution in a subset of publications about Computer Networks in DBLP dataset. The fitting parameters and errors are shown in Table 4. One can see that the new MC model reduces

Table 4. Fitting parameters and errors in social datasets. MC is the fitting error of our model, PL is the fitting error of using power-law, $PL-C$ is the fitting error of using power-law with expoenetial cutoff, and $SPL-C$ is the error of shifted power-law with geometric cutoff.

Dataset	L	U	exponent	MC	PL	$PL-C$	$SPL-C$
DBLP	18	250	−2.51	0.10	0.58	0.56	0.10
Networking	4	30	−2.78	0.07	0.57	0.34	0.13
Facebook	10	25	−1.42	0.02	0.25	0.37	0.03
Twitter	6	43	−2.44	0.01	0.13	0.26	0.02

the fitting errors by up to 95%.

Implications to social network analysis: In social networks, the distribution of the numbers of friends or coauthors that each people has is an important indicator of the health of the networks. Typically, the more evenly distributed the network is (i.e., the more number of friends each people has), the healthier (i.e., more desirable) it is. In the MC model, this corresponds to a smaller L, as the exponent in the power-law region γ is proportional to L, hence a smaller L implies a flatter slope, which means a flatter distribution. However, as proven in the MC model, a smaller L also means a smaller growth rate of the network. Hence, there is a tradeoff in obtaining a healthier distribution and a faster growth of the network by changing L. In practice, an administrator of a social network can control this L in various ways. For example, the administrator can introduce subsidies or promotions to lure the recruitment of new members, hence increasing L, or introduce fees for joining the network, hence reducing L, so as to obtain a desired tradeoff level.

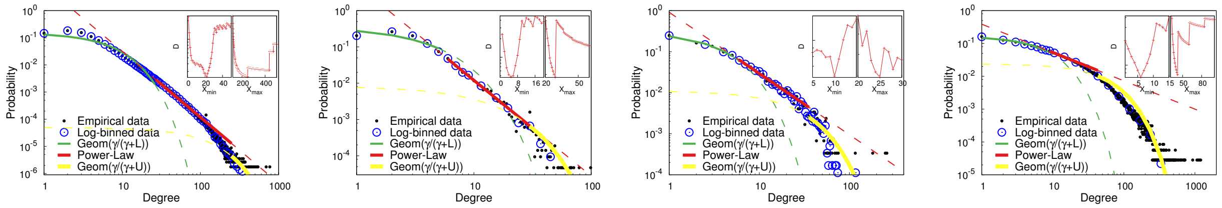

(a) DBLP coauthorship (b) DBLP: Networking (c) Facebook (d) Twitter

Figure 4. Probability $P(x)$ versus node degree x on a double logarithmic scale in social network datasets. The node degree represents the number of coauthors, friends, or followers for the three datasets, respectively. The inner figures show the values of Kormogorov-Smirnov test vs. X_{min} and X_{max}. $\mathscr{L}$ and $\mathscr{U}$ are selected from X_{min} and X_{max} to minimize the KS-test distance value.

S10.2 Vehicular networks

Table 5 shows two vehicular networks datasets[30–32], which are used in our verification and analysis.

MC model of Vehicular networks: In vehicular networks, vehicles are considered as nodes. When a vehicle $v1$ is within the communication range r with another vehicle $v2$, a link is built between $v1$ and $v2$ and the contact counts (i.e., node degrees) of both vehicles increase by 1. A vehicle's degree is related to the area it locates. For those vehicles located in a quiet zone, there are only a few vehicles around, so their node degrees are low (i.e., degrees $< L$). These vehicles move freely and have an equal probability to meet each other. On the other hand, a vehicle will have a higher degree if it is closer to the crowded areas (e.g., downtowns or hot scenic points). When those vehicles get closer to such areas, they will meet more vehicles with higher probabilities. Vehicles located in such areas may be able to communicate with most vehicles in the areas and therefore can link to these vehicles with an equal probability.

Table 5. Vehicular Datasets.

Dataset	Date	Duration	# of vehicles
Rome Taxi[30]	Feb 01, 2014	30 days	316
Beijing Taxi[31,32]	Feb. 02, 2008	7 days	10,336

Table 6. Fitting parameters and errors in vehicular networks datasets. MC is the fitting error of our model, PL is the fitting error of using power-law, $PL-C$ is the fitting error of using power-law with expoenetial cutoff, and $SPL-C$ is the error of shifted power-law with geometric cutoff.

Dataset	L	U	exponent	MC	PL	$PL-C$	$SPL-C$
Rome	6	21	-1.20	0.03	0.36	0.15	0.10
Beijing	9	20	-3.19	0.06	0.54	0.11	0.08

Empirical Data and Fitting Results:

Fig. 5 shows the distributions of contact counts for Rome and Beijing taxi datasets. The fitting parameters and errors are shown in Table 6. One can see that the proposed MC model reduces the fitting errors by 23% - 90%. One can also observe a

similar trend that all the data include three phases. However, the fitting is not as good as that in citation and social networks. This is because, in vehicular networks, vehicles not only *build* links with other vehicles (when entering the communication ranges), but also *break* some links (when leaving the communication ranges).

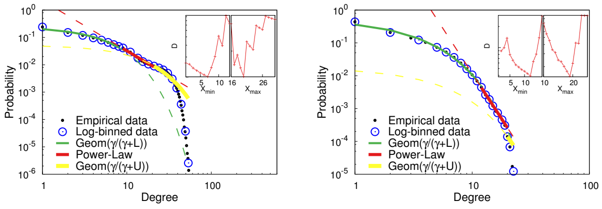

(a) Rome (b) Beijing

Figure 5. Probability $P(x)$ versus the node degree x on a double logarithmic scale in vehicular networks datasets. The node degree represents the contact counts. The inner figures show the values of Kormogorov-Smirnov test vs. X_{min} and X_{max}.

Implications to vehicular network design: The MC model can be used to design a better routing scheme for Delay-Tolerant Networking (DTN), such as vehicular networks. With the intermittent connections in DTN, one of the main challenges is how to select nodes to forward data in order to improve the reachability and throughput. Existing routing schemes rely on exchanging information when two nodes meet, and predicting if the node will get closer to the destination node in the future. These schemes however have two main disadvantages. First, the current prediction is usually based on the exponential or power-law model, which has been shown to have larger prediction errors above. Second, when two nodes meet, they need to exchange the complete or summarized history, which can occupy lots of bandwidths, especially when the intra-connection time is short. With the new MC model, one can calculate the probability directly according to the derived close-form formula, in which vehicles only need to exchange the simple information of their current node degrees.

References

1. Amaral, A., Scala, A., Barthélémy, M. & Stanley, H. Classes of small-world networks. *Proceedings of the National Academy of Sciences* **97**, 11149–11152 (2000).
2. Barabási, A.-L. *et al.* Evolution of the social network of scientific collaborations. *Physica A* **311**, 590–614 (2002).
3. Leskovec, J., Kleinberg, J. & Faloutsos, C. Graph evolution: Densification and shrinking diameters. *ACM Transactions on Knowledge Discovery from Data* **1** (2007).
4. Pham, T., Sheridan, P. & Shimodaira, H. Joint estimation of preferential attachment and node fitness in growing complex networks. *Scientific Reports* **6** (2016).
5. Dorogovtsev, S. N., Mendes, J. & Samukhin, A. Structure of growing networks with preferential linking. *Phys. Rev. Lett.* **85**, 4633 (2000).
6. Godreche, C., Grandclaude, H. & Luck, J. Finite-time fluctuations in the degree statistics of growing networks. *J. of Stat. Phys.* **137**, 1117–1146 (2009).
7. Albert, R. & Barabási, A.-L. Topology of evolving networks: local events and universality. *Phys. Rev. Lett.* **85**, 5234–5237 (2000).
8. Bianconi, G. & Barabási, A.-L. Bose-einstein condensation in complex networks. *Physical Review Letters* **86**, 5632–5635 (2001).
9. Bianconi, G. & Barabási, A.-L. Competition and multiscaling in evolving networks. *EPL (Europhysics Letters)* **54**, 436 (2001).
10. Krapivsky, P. L., Redner, S. & Leyvraz, F. Connectivity of growing random networks. *Phys. Rev. Lett.* **85**, 4629–4632 (2000).
11. Medo, M., Cimini, G. & Gualdi, S. Temporal effects in the growth of networks. *Phys. Rev, Lett.* **107**, 238701 (2011).
12. Bauke, H., Moore, C., Rouquier, J. & Sherrington, D. Topological phase transition in a network model with preferential attachment and node removal. *The European Physical Journal B* **83**, 519–524 (2011).
13. Goshal, G., Chi, L. & Barabási, A.-L. Uncovering the role of elementary processes in network evolution. *Scientific Reports* **3**, 1–8 (2013).

14. Moore, C., Ghoshal, G. & Newman, M. E. J. Exact solutions for models of evolving networks with addition and deletion of nodes. *Phys. Rev. E* **74**, 036121 (2006).

15. Eom, Y.-H. & Fortunato, S. Characterizing and modeling citation dynamics. *PloS one* **6**, e24926 (2011).

16. Papadopoulos, F., Kitsak, M., M. Ángeles Serrano, M. B. & Krioukov, D. Popularity versus similarity in growing networks. *Nature* **489**, 537–540 (2012).

17. Xie, Z., Ouyang, Z., Zhang, P., Yi, D. & Kong, D. Modeling the citation network by network cosmology. *PloS one* **10**, e0120687 (2015).

18. Yule, G. U. A mathematical theory of evolution, based on the conclusions of dr. j. c. willis, f.r.s. *Philosophical Transactions of the Royal Society of London Series B* **213**, 21–87 (1925).

19. Simon, H. A. On a class of skew distribution functions. *Biometrika* **42**, 425–440 (1955).

20. Erdös, P. & Rényi, A. On random graphs i. *Publicationes Mathematicae* **6**, 290–297 (1959).

21. Callaway, D. S., Hopcroft, J. E., Kleinberg, J. M., Newman, M. E. J. & Strogatz, S. H. Are randomly grown graphs really random. *Phys. Rev. E* **64** (2001). 041902.

22. Deng, W., Li, W., Cai, X. & Wang, Q. A. The exponential degree distribution in complex networks: Non-equilibrium network theory, numerical simulation and empirical data. *Physica A* **390**, 1481 – 1485 (2011).

23. Hauer, J., Demeure, C. & Scharf, L. Initial results in prony analysis of power system response signals. *IEEE Transactions on Power Systems* **5**, 80 –89 (1990).

24. Newman, M. *Network: An Introduction* (Oxford University Press, 2010).

25. Milojević, S. Power law distributions in information science: Making the case for logarithmic binning. *Journal of the American Society for Information Science and Technology* **61**, 2417–2425 (2010).

26. Tang, J. *et al.* Arnetminer: Extraction and mining of academic social networks. In *Proceedings of the 14th ACM SIGKDD International Conference on Knowledge Discovery and Data Mining*, KDD '08, 990–998 (2008).

27. Leskovec, J. & Krevl, A. SNAP Datasets: Stanford large network dataset collection (2014).

28. Mcauley, J. & Leskovec, J. Discovering social circles in ego networks. *ACM Transactions on Knowledge Discovery from Data* **8**, 4:1–4:28 (2014).

29. Leskovec, J. & Mcauley, J. J. Learning to discover social circles in ego networks. In Pereira, F., Burges, C., Bottou, L. & Weinberger, K. (eds.) *Advances in Neural Information Processing Systems 25*, 539–547 (Curran Associates, Inc., 2012).

30. Bracciale, L. *et al.* CRAWDAD dataset roma/taxi (v. 2014-07-17). Downloaded from http://crawdad.org/roma/taxi/20140717 (2014).

31. Yuan, J. *et al.* T-drive: Driving directions based on taxi trajectories. In *Proceedings of the 18th SIGSPATIAL International Conference on Advances in Geographic Information Systems*, GIS '10, 99–108 (ACM, New York, NY, USA, 2010).

32. Yuan, J., Zheng, Y., Xie, X. & Sun, G. Driving with knowledge from the physical world. In *Proceedings of the 17th ACM SIGKDD International Conference on Knowledge Discovery and Data Mining*, KDD '11, 316–324 (ACM, New York, NY, USA, 2011).
